# Neuro4PD: An Initial Neurorobotics Model of Parkinson's Disease

**DOI:** 10.3389/fnbot.2021.640449

**Published:** 2021-07-01

**Authors:** Jhielson M. Pimentel, Renan C. Moioli, Mariana F. P. de Araujo, Caetano M. Ranieri, Roseli A. F. Romero, Frank Broz, Patricia A. Vargas

**Affiliations:** ^1^Edinburgh Centre for Robotics, Heriot-Watt University, Edinburgh, United Kingdom; ^2^Bioinformatics Multidisciplinary Environment, Digital Metropolis Institute, Federal University of Rio Grande do Norte, Natal, Brazil; ^3^CCS, Federal University of Espírito Santo, Vitória, Brazil; ^4^ICMC, University of São Paulo, São Paulo, Brazil

**Keywords:** neurorobotics, basal ganglia, motor control, Parkinson's disease, motor cortex, robot controller

## Abstract

In this work, we present the first steps toward the creation of a new neurorobotics model of Parkinson's Disease (PD) that embeds, for the first time in a real robot, a well-established computational model of PD. PD mostly affects the modulation of movement in humans. The number of people suffering from this neurodegenerative disease is set to double in the next 15 years and there is still no cure. With the new model we were capable to further explore the dynamics of the disease using a humanoid robot. Results show that the embedded model under both conditions, *healthy* and *parkinsonian*, was capable of performing a simple behavioural task with different levels of motor disturbance. We believe that this neurorobotics model is a stepping stone to the development of more sophisticated models that could eventually test and inform new PD therapies and help to reduce and replace animals in research.

## 1. Introduction

This paper describes the first steps toward the creation of a novel neurorobotics model of Parkinson's Disease (PD)^1^. PD is characterised by a disruption of the Basal Ganglia (BG) circuitry, which is composed of a set of nuclei linked to the thalamus and cortex in our brain. PD affects the modulation of movement (Bear et al., 1996; Jankovic, 2008) apart from other symptoms (Girotti et al., 1986; Goodarzi and Ismail, 2017). This neurodegenerative disease affects more than 3% of people over 65 years old, with figures set to double in the next 15 years (Dorsey et al., 2007; Rizek et al., 2016). There is still no cure, and therapies rely heavily on a few incomplete computational models of PD, which were created based on animal models (Humphries et al., 2018).

Computational models developed to date often neglect the behavioural effects of physical bodies interacting with the environment, such as sensorimotor regularities. Apart from that, due to experimental limitations, the data used to tune these models are normally from only a couple of relevant brain regions, collected in different behavioural contexts, possibly from various animal lineages and vivaria. As a consequence, it is largely neglected that animal sensorimotor mechanisms and PD symptoms result from self-organised dynamic processes. The misinterpretation of these processes, which emerge out of the brain-body-environment interactions, might undermine PD computation models' fidelity.

In this paper, we embed a biophysically plausible computational model specifically tuned for the 6-hydroxydopamine rat model of PD in a real humanoid robot that is engaged in a simple behavioural task. The computational model represented by the basal ganglia-thalamus-cortex system has been fully tuned and validated with published data (Izhikevich and Edelman, 2008; Van Albada and Robinson, 2009; Robinson and Kim, 2012; Kerr et al., 2013; Kumaravelu et al., 2016). We also created a sensorimotor loop with biologically informed constraints that can modulate human's body inherent oscillatory phenomena (i.e., central oscillators; Burkhard et al., 2002). This is an important step toward advancing our knowledge about PD beyond that obtained from anatomical and physiological studies (Lang and Lozano, 1998; Kalia and Lang, 2015). The main contributions of this paper are: (i) an embedded computational composite model of PD in a real humanoid robot and (ii) the reproduction of abnormal PD motor stimulation based on cortical dynamics via the modulation of central oscillators. We expect new insights into PD by future studies conducted using our proposed model.

This paper is organised as follows: section 2 introduces the Basal Ganglia-Thalamus-Cortex system focusing on its main motor pathways. Section 3 presents the main computational models of PD. Section 4 discusses related works on neurorobotics approaches to neurodegenerative diseases. Section 5 proposes a novel neurorobotics model of PD. Section 6 presents the methods and experimental setup, including the humanoid robot used. Section 6 shows the results and section 7 presents the discussion. Section 8 draws conclusions and proposes future work.

## 2. The Basal Ganglia-Thalamus-Cortex System

**Figure 1:**
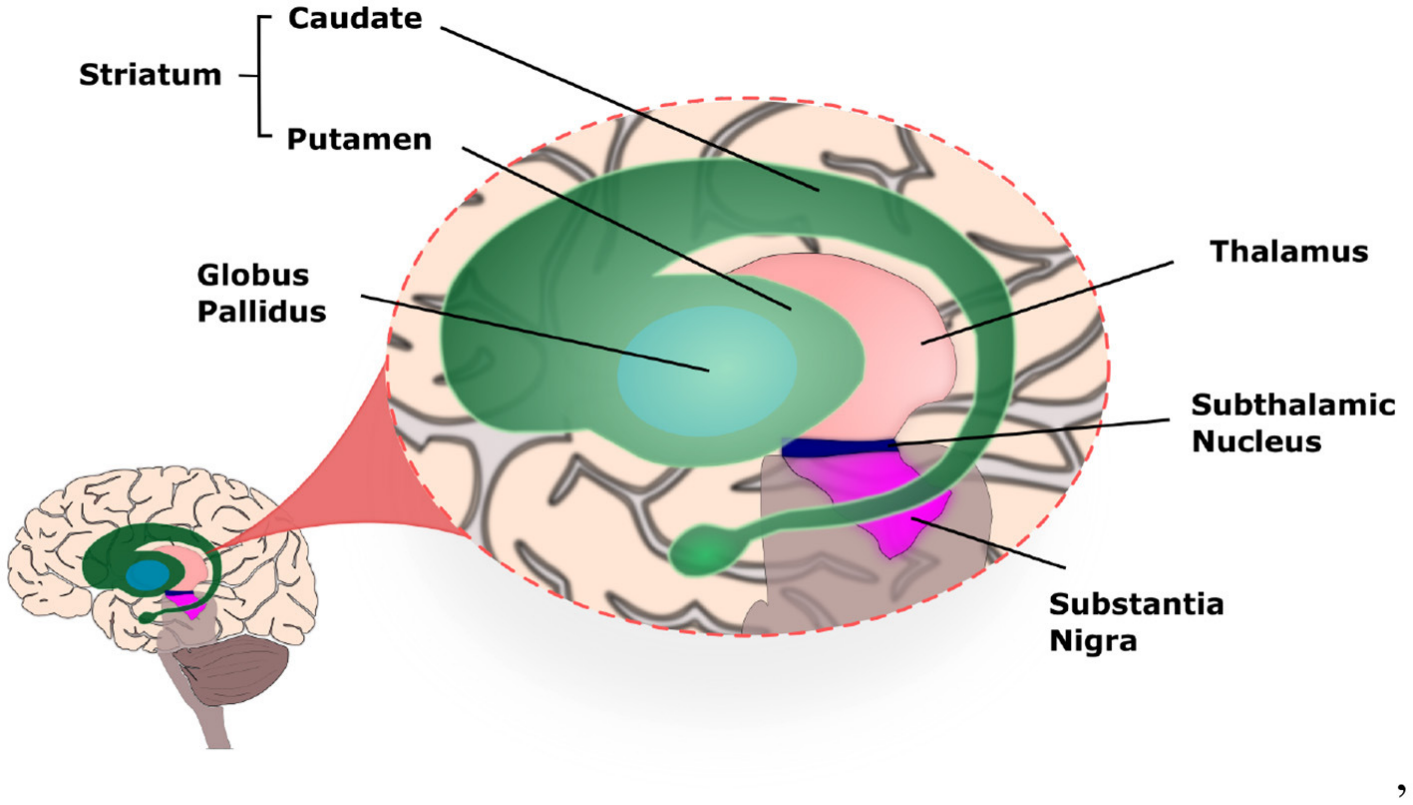
Illustration of the basal ganglia highlighting the location of each nucleus within the human brain.

The basal ganglia (BG) circuitry is a set of interconnected nuclei including the Striatum (caudade and putamen), Globus Pallidus pars interna and pars externa (GPi and GPe), SubThalamic Nucleus (STN), and Substantia Nigra pars reticulata and pars compacta (SNr and SNc; Figure 1). Together with the thalamus (Haber and Calzavara, 2009) and cerebral cortex, they form the Basal Ganglia-Thalamus-Cortex System (BG-T-C System), a highly organised network formed by parallel sensorimotor, associative, and limbic loops, involved, respectively, with movement control, cognition, and processing of reward and emotions (Obeso et al., 2008; Redgrave et al., 2010; Galvan et al., 2015).

### 2.1. The Sensorimotor Loop

**Figure 2:**
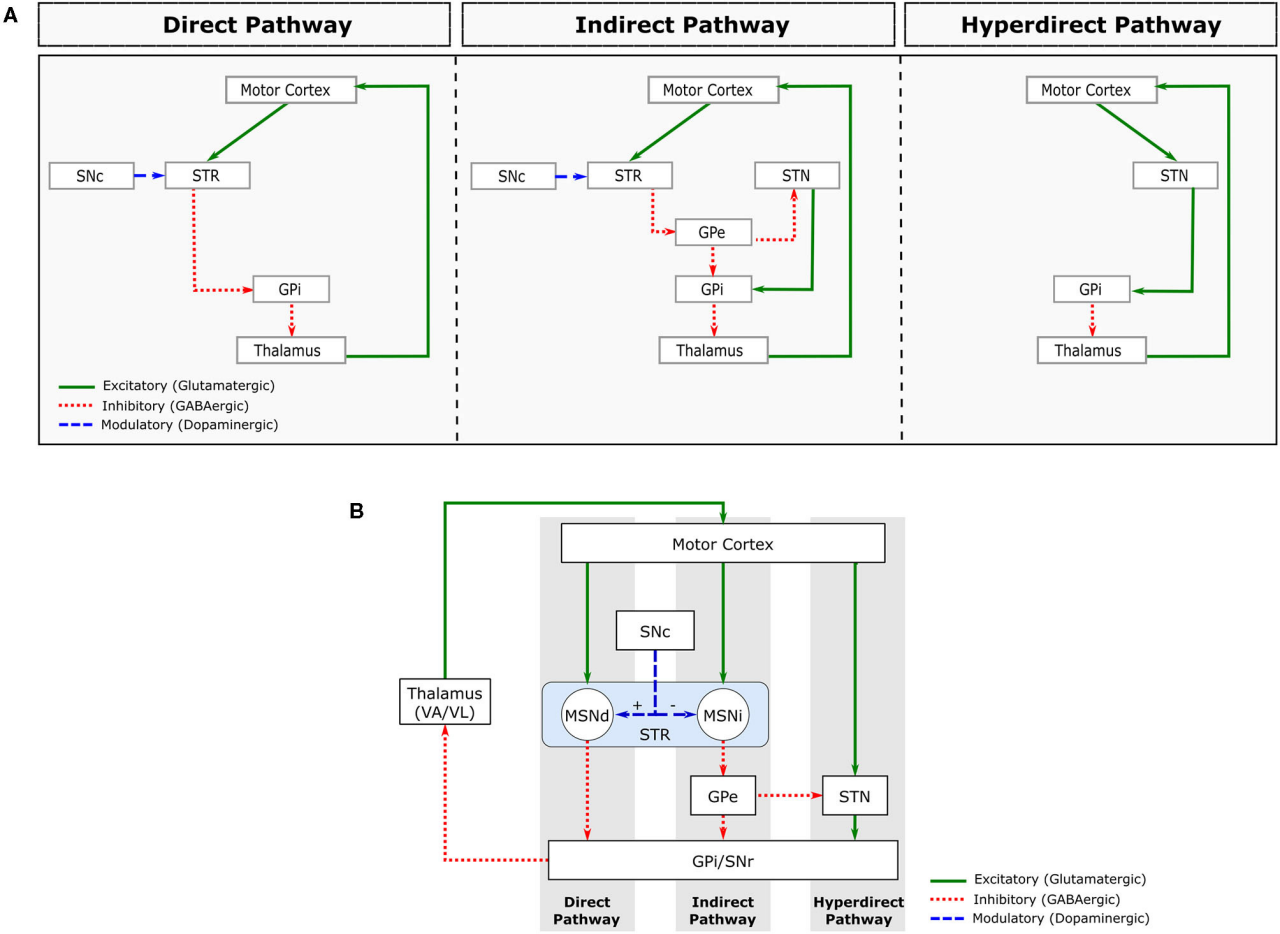
Major pathways comprising the sensorimotor loop. **(A)** Schematic representation of the direct (left), indirect (centre) and hyperdirect (right) pathways. **(B)** Combined representation of these pathways. STR, striatum; MSNd, medial spiny neurons of the direct pathway; MSNi, medial spiny neurons of the indirect pathway; SNc, substantia nigra pars compacta; GPe, globus pallidus pars externa; GPi, globus pallidus pars interna; STN, subthalamic nucleus; VA/VL, ventroanterior and ventrolateral thalamic nuclei.

Movements in our body are controlled by a *sensorimotor loop* (SML), which is comprised by somatotopically organised^2^ excitatory projections from cortical motor areas and primary somatosensory cortex to the BG input nuclei (Striatum and STN). Those in turn project to the motor regions of other BG nuclei. The BG output nuclei (GPi and SNr) then project to the Ventroanterior (VA) and Ventrolateral (VL) thalamic nuclei, which then project back to the motor regions of the cortex (Obeso et al., 2008). These connections within the sensorimotor loop are mediated by neurons that establish either excitatory or inhibitory synapses, which are mediated, respectively, by the neurotransmitters glutamate and Gamma-AminoButyric Acid (GABA) (Figure 2).

About 90% of Striatum cells are Medium Spiny Neurons (MSNs), which are GABAergic inhibitory cells that receive excitatory glutamatergic projections from the cortex and thalamus. The MSN projections form 2 distinct circuits, called *direct* and *indirect* pathways (Obeso et al., 2008; Lanciego et al., 2012; Figure 2A).

The MSNs from the *direct* pathway project directly to the BG output nuclei (GPi/SNr), while the MSNs from the *indirect* pathway project to the GPe. GPe is a BG nucleus containing GABAergic inhibitory neurons that project to the GPi/SNr directly and indirectly via the STN. In addition to GABAergic projections arriving from GPe, the STN receives excitatory glutamatergic projections directly from the cerebral cortex. This circuit is often called the *hyperdirect* pathway (Nambu et al., 2002; Figure 2A).

The STN is mainly composed of glutamatergic neurons that send excitatory projections to BG output nuclei (GPi/SNr). Both BG output nuclei (GPi and SNr) contain inhibitory GABAergic neurons that fire tonically (i.e., with a sustained firing frequency), inhibiting the VA/VL thalamic nuclei. The *direct* pathway facilitates movement by inhibiting the tonic activity of GPi/SNr, inducing a pause on their neuronal firing. The *indirect* and *hyperdirect* pathways, on the other hand, inhibit movement by increasing the tonic inhibitory activity of the BG inhibitory outputs.

### 2.2. The Parkinsonian Conditions

Dopamine (DA) is another neurotransmitter involved in the sensorimotor loop. DA Projections from SNc to striatum modulate the activity of the *direct* and *indirect* pathways by regulating MSN excitability (Figure 2B). Specifically, dopamine excites MSN neurons in the *direct* pathway and inhibits MSN neurons in the *indirect* pathway. The combined effect of DA on *direct* and *indirect* pathways, therefore, leads to a decrease in GPi/SNr activity, decreasing the inhibition of the thalamocortical projection neurons. Therefore, the main effect of DA release in Striatum is movement facilitation.

In PD, there is a progressive degeneration of SNc DA neurons. The depletion of striatal DA leads to a functional imbalance of BG circuitry, with enhanced activation of the *indirect* pathway and decreased activation of the *direct* pathway, resulting in an increase in GPi/SNr activity that hampers movement execution (Surmeier et al., 2014). In addition to these changes in firing rates, the *parkinsonian* conditions is also characterised by changes in firing patterns within each nucleus and amongst the structures of the BG-T-C system, such as increased synchrony between neighboring neurons, increased bursting activity and enhanced beta oscillatory activity (Galvan et al., 2015).

### 2.3. Tremor Symptom

The origin and mechanisms of motor symptoms of Parkinson's Disease (PD) are still under great dispute (McGregor and Nelson, 2019). In particular, there are several hypotheses for the origin of symptoms that are related to motor impairments but there is no consensus so far (Dovzhenok and Rubchinsky, 2012; Helmich et al., 2013). Most likely, a myriad of network interactions lead to motor disruptions that are observed in PD, including structures beyond the basal ganglia networks like the cerebellum. Traditionally, it is assumed that the basal ganglia and cerebellum are anatomically separated but interfaced via cortical networks. Please refer to Mori et al. (2016) for a more comprehensive discussion on the role of other structures.

Parkinson's tremor symptom is an involuntary motion, in particular on the upper limbs, with frequency of 4–6 Hz and high amplitude along a voluntary movement or at rest (Guyton and Hall, 2001; Haeri et al., 2005). A few previous works hypothesize that the dopamine depletion in the BG-T-C system may lead to the occurrence of the tremor symptom (Dovzhenok and Rubchinsky, 2012; Helmich et al., 2012; Cagnan et al., 2014). However, recent evidences from clinical, neuroimaging and postmortem studies link essential tremor to cerebellar dysfunction (Louis et al., 2006; Mirdamadi, 2016; Louis, 2018; Louis and Faust, 2020). The authors hypothesize that the cerebellum influenced by dopamine depletion may also contribute to the enhancement of the tremor. Nonetheless, the origin of the symptom is yet a mystery.

According to Burkhard et al. (2002), there are central oscillators that are composed of oscillatory components involving neuronal networks capable of generating a naturally occurring neural oscillation. These central oscillators synchronise cerebral function in brains under both conditions (*healthy* and *parkinsonian*). However, it is not very clear which structures of the brain are involved in the generation of these oscillatory patterns. Although the physiological function of those structures are yet unknown, the authors consider them to be directly related to motor control and tremor iythn PD. Based on experiments with healthy subjects executing voluntarily simulated tremor and data from pathological tremors recorded from patients with PD and essential tremor, the authors noticed the influence of the central oscillators on motor tasks.

Based on PD patients with electrode implantation, Du et al. (2018) observed evidences of oscillatory patterns in different regions of the brain, like STN, GPi, and ventral intermediate thalamus (Vim). They named the cells oscillatory neurons and associated them to the tremor symptom. In a different study, using a helmet-shaped 122-channel whole-head neuromagnetometer (Neuromag^*TM*^), Pollok et al. (2004) concluded that the same brain areas are involved in voluntary tremor as in parkinsonian resting tremor. Based on the acquired data, the authors considered that pathological tremors might be based on a physiological pre-existing cerebral oscillatory network. Unfortunately, more details on those oscillatory neurons are yet unknown.

## 3. Computational Models of Parkinson's Disease

At present, there are only a few computational models built by different research groups to support distinct investigations on motor and cognitive deficits of PD. In fact, neuroscientists have been using them to improve their understanding of motor symptoms associated to neural disorders like PD and many others (Cohen et al., 2002; O'Donnell and Wilt, 2006; Sarbaz et al., 2007; Schroll and Hamker, 2013; Sanger, 2018; Pena et al., 2020). In particular for PD, those models became an ally on a search for new treatments and therapies (Humphries et al., 2018), and also to enhance the efficacy of well-known treatments like Deep Brain Stimulation (DBS) (Rubin and Terman, 2004; Humphries and Gurney, 2012; Lu et al., 2020).

Some of the computational models of PD are built to support hypothesis on the cause of motor impairments. For instance, Pavlides et al. (2015) explains the proposed mechanisms for an anomalous increase in beta oscillations within the BG nuclei. Other models are built to investigate not the symptoms but the origin of the disease, as presented in Muddapu et al. (2019). The authors designed a model to explore the progressive and inexorable loss of dopaminergic cells in the SNc. In some cases, computational models of PD are designed inspired by the BG nuclei alone (Gurney et al., 2004; Merrison-Hort et al., 2017) while others build a more complex system incorporating the entire sensorimotor loop (Kumaravelu et al., 2016) (Figure 2B).

**Figure 3:**
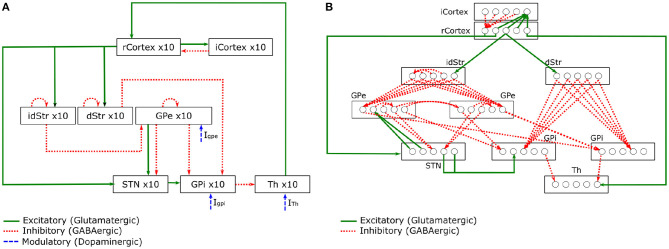
Schematic of the BG-T-C System computational model adapted from Kumaravelu et al. (2016). **(A)** Shows connections within the network. **(B)** Details of synaptic connections within the network model. Each rcortical neuron receives excitatory input from one TH neuron and inhibitory input from four randomly selected icortical neurons. Each icortical neuron receives excitatory input from four randomly selected rcortical neurons. Each dStr neuron receives excitatory input from one rcortical neuron and inhibitory axonal collaterals from three randomly selected dStr neurons. Each idStr neuron receives excitatory input from one rcortical neuron and inhibitory axonal collaterals from four randomly selected idStr neurons. Each STN neuron receives inhibitory input from two GPe neurons and excitatory input from two rcortical neurons. Each GPe neuron receives inhibitory axonal collaterals from any two other GPe neurons and inhibitory input from all idStr neurons. Each GPi neuron receives inhibitory input from two GPe neurons and inhibitory input from all dStr neurons. Some GPe/GPi neurons receive excitatory input from two STN neurons, while others do not. Each TH neuron receives inhibitory input from one GPi neuron.

Kumaravelu et al. (2016) is one of the very few models that can incorporate the behavioural effects of physical bodies interacting with the environment. In other words, a model that replicates some of the sensorimotor loop mechanisms discussed in section 2.1, which are observed in animals during behavioural tasks. This computational model of PD focuses on the BG-T-C system of rats (Figure 3). The entire model was tuned using a collection of data from 6-OHDA lesioned rats. Their model also followed an extensive validation, demonstrating that it can replicate a wealth of experimental data.

The model is divided into brain regions: CorTeX (CTX), Striatum (Str), SubThalamic Nucleus (STN), Globus Pallidus externa (GPe), Globus Pallidus interna (GPi)^3^, and THalamus (TH). Each region includes 10 single compartment model neurons (Hodgkin-Huxley or Izhikevich neurons) interconnected with synapses to form a functional network. All model equations can be found in Kumaravelu et al. (2016). In Figure 3A, the connections present the excitatory and inhibitory relations between the CTX, BG, and TH. The well-know pathways (*direct, indirect*, and *hyperdirect*) presented in Figure 2 can be reproduced by this model. In Figure 3B, a diagram shows the synaptic connections between the CTX, BG, and TH. The pattern exhibited by those synapses was designed based on prior computational models (Rubin and Terman, 2004; So et al., 2012).

The two pathways that process signals through the BG have opposite net effects on thalamic target structures. In Boison and Masino (2016), the authors explain that the excitation of the *direct* pathway has the net effect of exciting thalamic neurons (which in turn make excitatory connections onto cortical neurons) and excitation of the *indirect* pathway has the net effect of inhibiting thalamic neurons (rendering them unable to excite motor cortical neurons). In other words, the *direct* pathway engages the movement behaviour while the *indirect* pathway inhibits it.

## 4. Neurorobotics and Neural Disorders

Neurorobotics is a field of research that focus on the embodiment of neural systems, like computational models of biological neural networks, in artificial software and machines. In 2005, a robotic salamander was presented as one of the first models designed to explore the interaction between brain, body and environment (Ijspeert et al., 2005, 2007). This interaction brought several discussions of the impact of external information on the dynamics of those neural networks.

For the last few decades, neurorobots have been applied to a wide range of domains (Kaplan, 2008; Krichmar, 2018; Li et al., 2019). For instance, inspired on the action selection mechanisms of the basal ganglia, Prescott et al. (2006) proposed to embed a neuronal model in a mobile agent to control its action selection during the performance of a robotic task. In a different context, Edvardsen et al. (2020) incorporated classes of neurons from hippocampus to design a navigation strategy in cluttered environments. Some other works have focused on the development of prosthetics to assist the locomotion of animals (von Zitzewitz et al., 2016) and even on the improvement of human-robot interaction by designing systems that control the levels of awareness in humanoid robots (Lindberg et al., 2017; Balkenius et al., 2018).

Brain-inspired models embedded in robots can be also an important tool for supporting studies on neural disorders. For instance, Yamashita and Tani (2012) investigated psychiatric disease symptoms, including fictive perception, altered sense of self, and unpredictable behaviour, by embedding a neuronal model containing schizophrenia-like deficits in a humanoid robot engaged in a task that demanded interaction with the environment via object manipulation. Oota et al. (2018) investigated early onset symptoms of abnormal motor coordination in rats using a soft neurorobotics suit that provides integrated cognitive and physical interventions. For further discussion on neurorobotics models of neurological disorders, please check the mini review by Pronin et al. (2021).

Most of those works search for a deeper understanding of motor and cognitive symptoms by investigating the dynamics of human and animal brains. However, neural models are yet considerated to be abstraction since they can not replicate the same level of complexity observed in real brains. The most advanced projects modeling the biophysical characteristics of human brain can only perform a reduced number of brain cells (4 million point neurons and 31,000 detailed neurons) on High Performance Computing (HPC) clusters (Chi, 2016; Makin, 2019). Besides, in order to build more realistic neural model, live animals, like monkeys and rats, are used in experiments under specific scenario for data collection. In each experiment with a single subject, the activity of only a few regions of the brain can be collected. To acquire multiple data from different regions, it would demand several invasive surgeries in the same animal which is not ethical. Usually, models of specific regions are built upon data from several animals and, in some cases, even different species. Therefore, neural disorders are usually built upon sophisticated but simplified computational models of specific regions of the brain.

In a PD scope, to the best of our knowledge, sophisticated computational models, such as the one proposed by Kumaravelu et al. (2016), have never been embedded in a robot as a mechanism to understand the pathology, for diagnosis, or even to inform new treatments or therapies. Besides, Kumaravelu et al. (2016) describe brain structures that are important for the formation of sensorimotor loops, like BG, TH, and CTX.

Wang et al. (2010) designed computational models of Huntington's disease (HD) and PD based on leaky integrate-and-fire neurons, and embedded them in a simple Lego robot (Klassner and Anderson, 2003). By integrating the computational models to the robot's controller, they investigated an action selection mechanism and compared the robot's pattern in changing behaviours. The computational models created are composed of a BG type structure that is updated by sensory information in order to select the robot's behaviour (e.g., wandering, avoiding collision, and dancing; Wang et al., 2007, 2010). In comparison to the Kumaravelu et al. (2016) model, the model from Wang et al. (2007, 2010) provides less biophysical details of structures involved in HD and PD, which limits the generalisation of neural and behavioural dynamics reported.

Using a completely different approach, Kulam et al. (2011) implemented a model inspired only by the BG dynamics to reproduce PD symptoms, like bradykinesia, in a robotic arm. The authors used reinforcement learning to train their model and reproduce the desired symptoms. But, they did not use an artificial neural network with spiking neurons to design the computational model of PD. The model was implemented as a control system in which the dopamine signal is related to the incremental changes in error between the target position and the position of the end-effector of the arm. A biophysical computational model of the BG nuclei was not incorporated and, as a consequence, it may not be the most adequate tool to support neuroscientists in their investigations of PD.

To date and to the best of our knowledge, there are no biologically plausible neurorobotics models capable of reproducing characteristic symptoms of PD, like bradykinesia/akinesia, tremor, and rigidity in real humanoid robots. The majority of those aforementioned works concentrate their investigations in either modeling the circuits of the brain or building robotic devices based on simple neuroscience concepts (Yamashita and Tani, 2012; Idei et al., 2017; Lewis and Cañamero, 2017). A true intersection between neuroscience and robotics is yet to be unveiled. Our work presents the first steps toward the creation of an embedded realistic computational model of PD in a humanoid robot. This new neurorobotics model has the potential to support researchers from different areas, like biologists, physicists, and neuroscientists, which might help to unravel the mysteries of PD.

## 5. Neuro4PD: Neurorobotics Model of Parkinson's Disease

In this section, we introduce a new neurorobotics model of PD that is comprised by a computational model of the disease, a humanoid robot, and a dedicated sensorimotor loop. Here we explain how the computational model of Kumaravelu et al. (2016) adapted by Romano et al. (2020) was embedded in our humanoid robot and how the devised model can be easily adapted to different behavioural tasks and applications.

### 5.1. The Computational Model

The chosen computational model of PD (Romano et al., 2020) is capable of artificially reproducing the biophysical features of the BG-T-C system, thus replicating the biological SML mechanisms discussed in section 3. The model was adapted from Kumaravelu et al. (2016) to an open-source Python package to facilitate the development, simulation, and analysis of our biological neuronal network using the NEURON simulator. The equations (summarised in Supplementary Table 1) describing the dynamics of each type of neuron were replicated without any change. Romano et al. (2020) modelled the same three conditions representing control (normal), 6-OHDA lesioned (PD), and 6-OHDA lesioned plus STN DBS in rats. In this work, we focused only on the *healthy* and *parkinsonian* conditions. Kumaravelu et al. (2016) explained how the changes in the synaptic conductance of specific neurons allowed them to replicate the loss of striatal dopamine neurons. In short words, the shift from healthy to PD conditions can be described as follow: decreasing the maximal M-type potassium conductance in direct and indirect MSN neurons (MSN firing dysfunction) from 2.6 to 1.5 *mS*/*cm*^2^; decreasing the maximal corticostriatal synaptic conductance (reduced sensitivity of direct MSN to cortical inputs) from 0.07 to 0.026 *mS*/*cm*^2^; and increasing the maximal GPe axonal collaterals synaptic conductance from 0.125 to 0.5 *mS*/*cm*^2^ (increase of GPe neuronal firing). More details of the implementation of each condition can be found in Kumaravelu et al. (2016).

To embed the computational model of PD in our humanoid robot, we first had to create the desired robot sensorimotor loop. Following the SML described in section 3, the robot should be able to get inputs from its sensory signals, incorporate them into the embedded computational model, and generate motor commands, which in turn will lead to new sensory inputs, thus closing the loop.

### 5.2. The Robot Sensorimotor Loop

It is clear from section 2.1, that the BG-T-C system and its communication pathways allow mammalians to produce motor responses based on sensory information thus giving rise to a sensorimotor loop. Hence, the interplay between those parts allows animals to make decisions based on internal and external stimuli.

One can also observe that the thalamus and the cortex form a closely coupled system (Saalmann and Kastner, 2011), where the thalamus transmits information from the environment to the cortex, and the cortex sends the output from multiple processing stages back to the thalamus. Based on this assumption, we decided to stimulate the thalamus based on visual cues and to read the output neuronal signals from the cortex to create our sensorimotor loop.

Inspired by the existence of central oscillators (section 2.3), the motor commands from cortical neurons are then combined and modulated by this inherent oscillatory phenomena (Burkhard et al., 2002). Similar modulation techniques have been observed in neuronal activity in animals and humans. For instance, Tomassini et al. (2017) investigated how the rhythm of different neuronal signals may modulate the perceptions of animals; and Ferguson and Cardin (2020) discussed how multiple input streams of cognitive, sensory or motor origin may modulate cortical gain. In our work, we investigated how the dynamics of the cortical neurons from our BG-T-C system under both *healthy* and *parkinsonian* condition may modulate the oscillatory signals from the central oscillators that might be the cause of the tremor symptom in PD patients. Hence, the final motor command is a result of an emulated signal which is able to simulate motor perturbation in the robot's upper limbs movements in *parkisonian* condition, as further explained on section 6.4.

**Figure 4:**
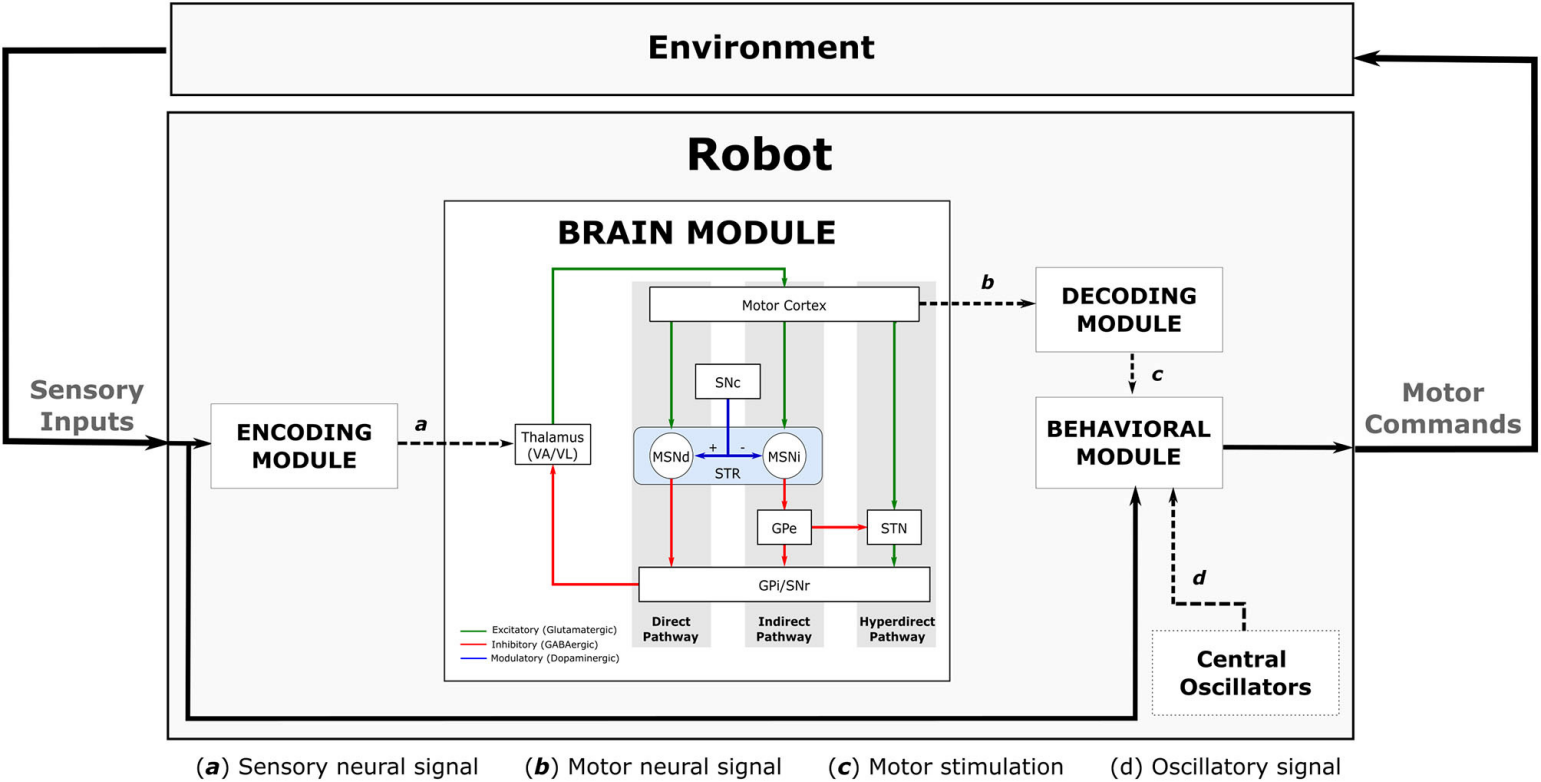
Full diagram showing the robot sensorimotor loop. The robot adjusts the sensory inputs from the environment through the Encoding Module (EM). The sensory information is then propagated throughout the Brain Module (BM) affecting the dynamics of the cortical neurons which are used to adjust the robot's movements. The dynamics of cortical neurons are decoded to motor stimulation by the Decoding Module (DM), then it is used to modulate the oscillatory signal received from the central oscillators inside the behavioural Module (BeM). The BeM then translates all these signals into motor commands with certain level of disturbance.

Our robot sensorimotor loop has four complementary modules (Figure 4): (i) the first module, Encoding Module (EM), is responsible for encoding environmental input into the BG-T-C system; (ii) the second module, Brain Module (BM), processes the input information respecting the dynamics of the embedded computational model of the brain; (iii) the third module, Decoding Module (DM), decodes the brain dynamics into motor stimulation; and (iv) the last module, Behavioural Module (BeM), translates the sensory inputs, the motor stimulation, and the oscillatory signals from the central oscillators into robot commands. Implementation details of each module will be discussed in section 6. The following subsections describe the modules as a generic framework which can be implemented in different ways without compromising the effectiveness of the entire sensorimotor loop.

#### 5.2.1. The Encoding Module

The encoding module focuses on the process of extracting information from the environment to stimulate the thalamic neurons in the BM. We designed the EM by transforming visual cues perceived by the robot into thalamic stimulation. For instance, if the robot detects a visual cue, a certain level of electric stimuli is applied directly to thalamic neurons, which in turns changes the dynamics of the entire brain module.

We used visual cues in our work, however, one can use any other type of sensory information, like voice commands and tactile inputs, or even a combination of those.

#### 5.2.2. The Decoding Module

The Decoding Module (DM) decodes the dynamics of cortical neurons of our embedded computational model into patterns that will be later translated into poses that will form the trajectory of the robot's limbs. Those poses allow the robot to perform pre-defined movements.

In this work, the DM extracts three features from the cortical neurons in order to produce motor signals: the firing rate (FR), the average of interspike intervals (ISIs), and the standard deviation of ISIs. Depending on the purpose of the neurorobotics model, any other set of features can be used. Also, different regions of the brain and different type of neurons could be more suitable.

#### 5.2.3. The Behavioural Module

**Figure 5:**
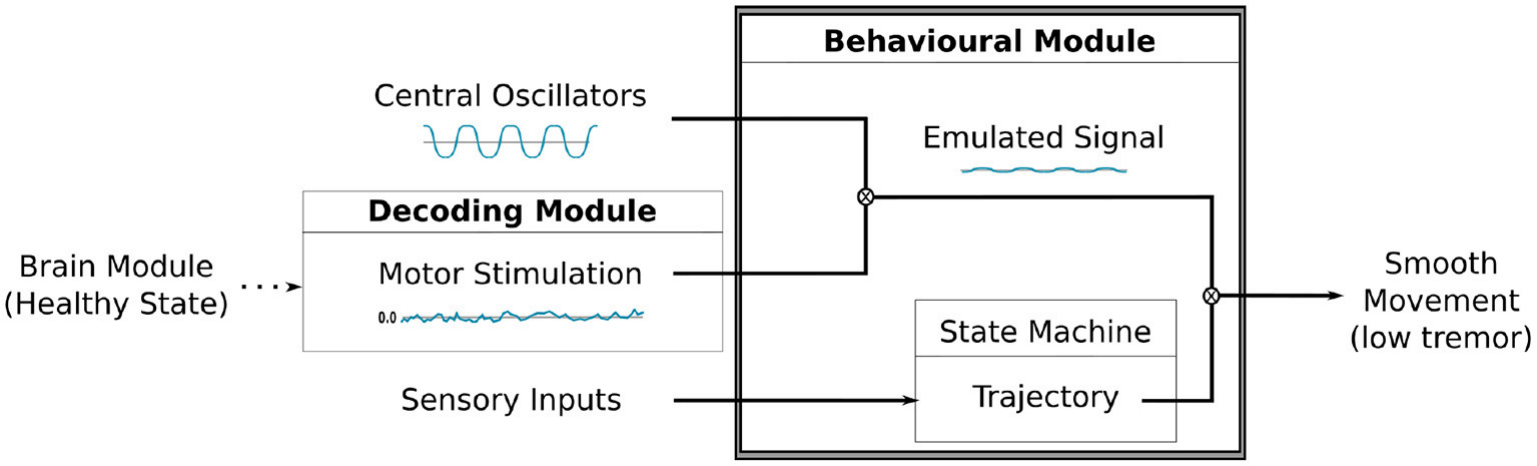
A diagram illustrating the modulation process between the motor stimulation produced by the DM and the oscillatory signal by the Central Oscillators. The resulting signal is combined to the trajectory, defined by our state machine, to finally produce the movement on the robot's arm.

This module is responsible for generating motor commands based on sensory inputs, motor stimulation and oscillatory signals created by the central oscillators. As depicted in Figure 5, the intensity or amplitude of the oscillatory pattern will be modulated by the motor stimulation produced by the DM. This gives rise to an “emulated signal,” which will adjust the poses defined by the state machine. During the performance of the motor tasks, each joint of the robot's arm will be able to receive updates of its state that might include some disturbance caused by the modulation of the oscillatory signals, thus closing the sensorimotor loop.

The BeM can reproduce all the robot's expected behaviours under both conditions of the embedded computational model, e.g., *heathy* or *parkinsonian*. We combined pre-defined poses with motor stimulation and an oscillatory pattern but different techniques could be used to produce the robot's trajectories. Also, it is important to highlight, this module can be adapted to a completely different behavioural task.

## 6. Methods

In this section we provide implementation details of our Neurorobotics model by presenting the behavioural task chosen for the SML experiments, the experimental scenarios, the robot architecture, and the software platforms used.

### 6.1. The Robot Architecture

**Figure 6:**
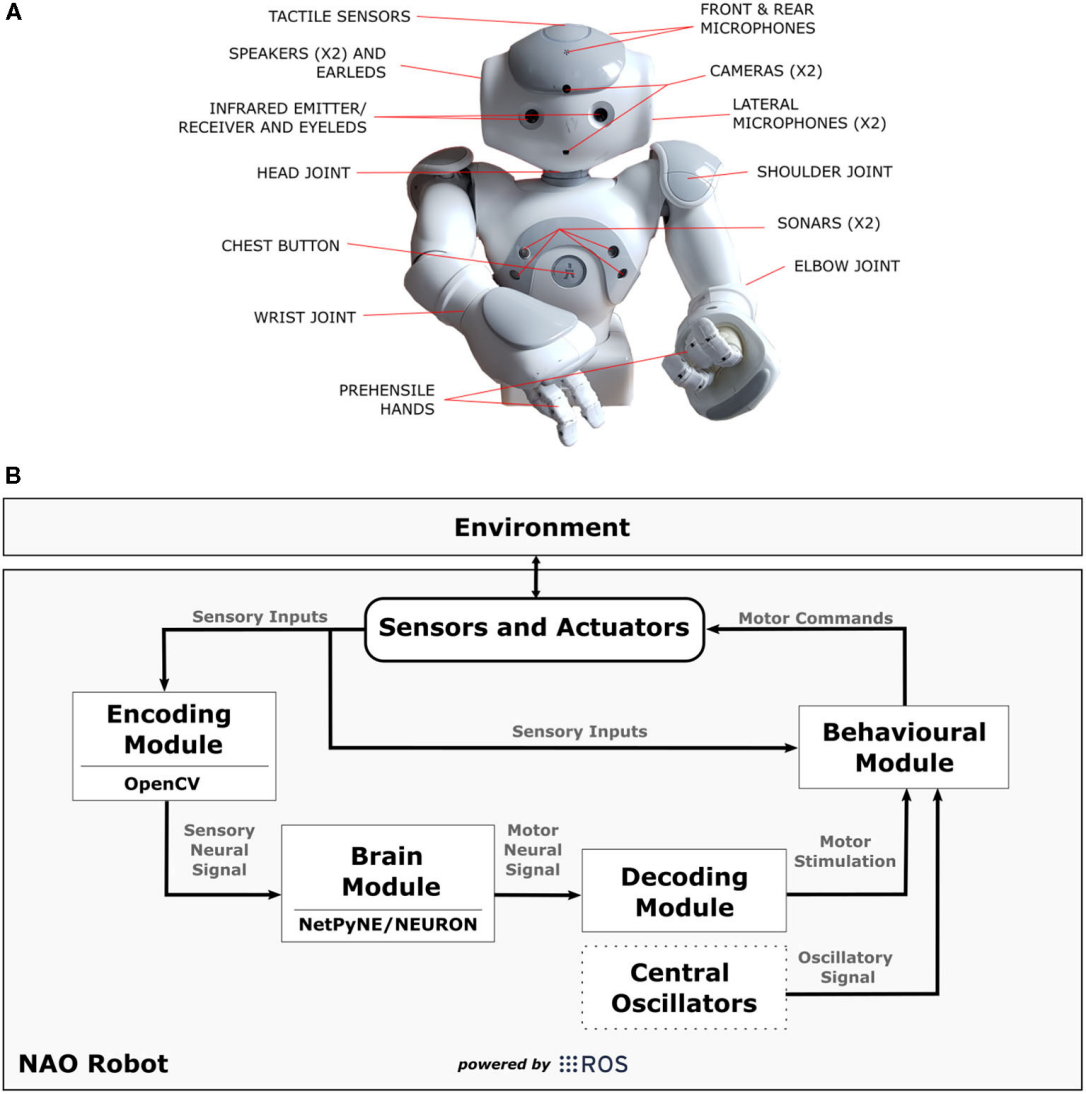
In **(A)**, technical illustration of NAO^T14^ robot indicating the position of sensors and actuators. **(B)** Shows a diagram illustrating the connections between the modules [Encoding Module (EM), Brain Module (BM), Decoding Module (DM), Behavioural Module (BeM)] and NAO robot in the SML. The modules represent different applications that complement each other during the task, and the arrows indicate the data flow. The Central Oscillators generate the oscillatory signals mimicking the inherent oscillatory patterns that are related to motor control and may be linked to *parkinsonian* tremor. ROS was mainly used as a platform to support communication between those elements and to interact with the robot by reading its sensors and sending commands to its actuators.

The robot architecture chosen for the experiments was the NAO^T14^ “torso only” robot (Gelin, 2018) (Figure 6A). NAO torso has a set of sensor and actuators like cameras, tactile sensors, and motors that allows the robot to move its upper limbs and interact with the environment. It is fully open and programmable and runs on NAOqi OS. Besides that, each arm of the robot has five degrees-of-freedom allowing it to reproduce several movements mimicking those of a human being.

We have implemented the four modules from our Neurorobotics Model (section 5) using Python programming language (Rossum, 1995) plus OpenCV (Bradski, 2000), NetPyNE/NEURON (Hines and Carnevale, 2001; Dura-Bernal et al., 2019), and ROS (Robot Operating System) (Cousins, 2010). The diagram in Figure 6B shows the connections between those modules [Encoding Module (EM), Brain Module (BM), Decoding Module (DM), Behavioural Module (BeM)]^4^ within NAO robot. It is important to mention that any other humanoid robot can be easily adjusted to this work if capable to communicate to our modules through ROS.

### 6.2. Behavioural Robot Task

**Figure 7:**
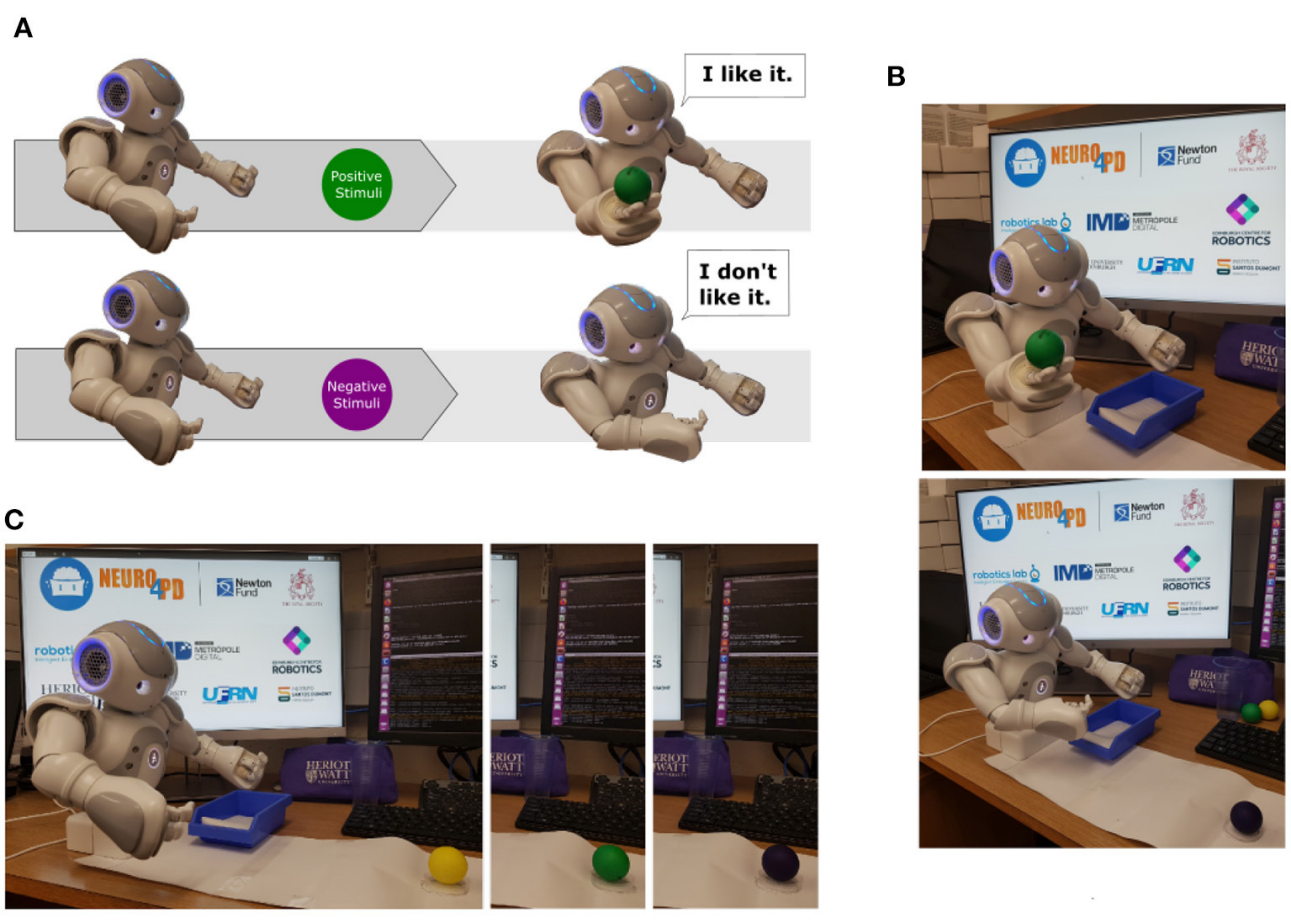
In **(A)**, two different destination poses based on distinct visual stimulus. The first one, the robot senses the object as a positive stimulus. It then extends its right arm and opens its hand to grasp the object. The second one, the robot senses the object as a negative stimulus and moves its right arm closer to its torso similar to a rejection behaviour. **(B)** Shows behavioural responses based on visual information. On the top image, the robot sensed a positive stimulus and activated the curiosity behaviour. On the bottom image, the robot sensed a negative stimulus and initiated the rejection behaviour. In **(C)**, the composed image presents the stage of the task when the robot senses each ball based on its colour: yellow, green, and purple.

The behavioural task chosen to assess the sensorimotor loop (section 2.1) consists of a simple visual/motor activity. Basically, the robot performs two different behaviours in response to distinct visual cues as illustrated in Figure 7A. If the robot senses an object as a positive stimulus, it extends its right arm and opens its hand to grasp the object. Otherwise, if the robot senses an object as a negative stimulus, it moves its right arm closer to its torso similar to a rejection behaviour.

The task was designed using simple movements that simplify further analyses of the robot's performances in both conditions, *healthy* and *parkinsonian*. Movements are possible by a continuous update of the robot's motor trajectories control with the desired motion. The motion was defined as destination poses, which were pre-defined with specific joints' angles. Here, we opted for a discrete set of motor trajectories that result from transitions of a state machine.

### 6.3. Experimental Scenarios

The experimental scenarios are composed of one humanoid robot (NAO^T14^), three balls with different colours (yellow, purple and green), and a computer to run ROS and NetPyNE/NEURON applications. We used the colour of the objects to trigger two different behaviours: curiosity and rejection to interact with an object (Nieuwboer et al., 2007) as presented in Figure 7B.

In short words, the behaviour task designed to support this work can be described as a sequence of interactions with the coloured balls by the robot. The main idea was to exploit our sensorimotor loop, which produces behavioural responses based on visual stimuli. In this work, when the task is initialised, the robot checks if there is any object ready to be sensed, as presented on Figure 7C. A small mark in front of the robot indicates the position where the objects are allocated so they can be easily detected. After the recognition of each object, the robot communicates the colour of the ball and, only then, initiates the related behaviour, rejection, or curiosity. A negative stimulus, indicated by the yellow or purple balls, produces the rejection behaviour while a positive one, represented by the green ball, produces the curiosity behaviour. After completing the last iteration, the robot communicates the end of the task and moves to its final pose.

In this work, we ran our experiments using only three iterations by the following order: yellow, green and purple. However, the number of iterations and the sequence of colours of the balls can be adjusted a priori any experiment. During the first 30 s of our experiment, the robot was initiated and none of the objects presented. This period allowed the robot to bring up all the sensors and actuators of the robot, and also to move the arms to their initial position. Then, each ball was allocated in front of the robot respecting the pre-defined sequence, which was the yellow ball at 30 s, followed by a green ball at 55 s, and a purple ball at 75 s after the beginning of the task. The duration of the entire task was 90 s. The time between those events allowed the robot to react to the objects by producing the expected behaviours.

#### 6.3.1. Experimental Scenario 01 (Negative Stimulus): Rejection Behaviour

If the robot detects either a purple or yellow ball, it refuses to play and moves the right arm^5^ toward its torso avoiding contact to the ball. In parallel, the robot communicates the decision of not interacting with the ball and asks for a new one. After rejecting the object, the robot moves back to its initial pose where it waits for the next iteration.

#### 6.3.2. Experimental Scenario 02 (Positive Stimulus): Curiosity Behaviour

The green ball represents a positive stimulus and it triggers the curiosity behaviour which is implemented as requesting the ball. Since the NAO robot (T14) presents some physical limitations that makes object grasping a challenge, it verbally asks someone to put the green ball directly on its hand by moving the right arm forward and opening the hands. Once the ball is grasped, the robot moves the arm toward a small container and drops the green ball in. Then, it moves back to the initial pose and waits for a new ball to be placed on the mark.

### 6.4. Environment Integration: NEURON, NetPyNE, and ROS

The computational model of PD embedded in the humanoid robot was adapted from Kumaravelu et al. (2016) by Romano et al. (2020) to make use of NetPyNE (Networks in Python and NEURON), a tool which provides a simplified interface to implement multiscale network models in the NEURON simulation environment (Hines and Carnevale, 2001). The simulator is a well-known tool that has been extensively used by neuroscientists due to its high reliability in terms of computational neuroscience.

The dynamics of the computational model brings a realism that is fundamental for the purpose of this work. The Hodgkin and Huxley, and Izhikevich neuron models allow the neurorobot to reproduce most of the neurocomputational dynamical features of specific neurons. For instance, the excitatory pattern from cortical neurons also known as regular spiking (RS). In this way, it is possible to generate equivalent patterns observed on real data, collected from animal experimentation, in response to injected pulses of DC current.

We decided to use the Robot Operating System (ROS) to create a bridge between NetPyNE and the robot. ROS is a free and open-source system that has grown out of a novel collaboration between industry and academia (Cousins, 2010). It provides mechanisms to communicate between different applications via topics and services. Thus, the model implemented on NetPyNE could be simulated in NEURON and, in parallel, communicate to other running packages besides the robot. In other to test the entire environment integration, we used Webots simulator (Michel, 2004) to run our experiments. Then, we moved to a real robot.

The neurorobotics model embedded in the robot was designed in subroutines that can be easily replaced without causing a chain of changes. In this way, it is more suitable for updates in the code and even new applications. For example, any module of the SML can be substituted to adapt to a new application. They have separate functionalities as described in section 5.2.

#### 6.4.1. Encoding Module

During the task, the robot captures images in high frequency by the camera attached to its head and transmits them to the first module (EM). The position of the object and robot are fixed for simplicity. The section of the images where the object is expected is then selected and its colour identified using the OpenCV library. Basically, we used hue values to group the pixels of the cropped image based on different ranges of colours. Then, the colour of the object is identified by checking the range which contains the highest number of pixels.

This information is used as stimulus to activate thalamic neurons in the BM. We defined two different levels of electrical stimuli based on the robot's behaviours (curiosity and rejection). When the first scenario (section 6.2) is identified, the sensory neural signal is set to its default stimulation, which is 1.2 mA (Kumaravelu et al., 2016). But, when the second scenario is spotted, it is updated to 3.5 mA (over stimulation). The stimulation is maintained until the robot detects a new object.

In this work, since the visual cues identified in this module are also requested later by the BeM, we decided to propagate them instead of the raw sensory inputs as presented in Figure 4. However, in a different and more complex behavioural task, the BeM might need access to the entire sensory inputs instead of only the visual cues. That is why we decided to connect the sensory inputs directly to the BeM in the diagram of the robot sensorimotor loop, as illustrated on Figure 4.

#### 6.4.2. Brain Module

Inside the BM, the stimuli received by the previous module (EM) are applied directly to thalamic neurons by modifying the stimulation parameters via commands of NetPyNE. These stimuli rapidly propagates the information to the rest of the network affecting the dynamics of the cortical neurons.

In this context, three features are extracted from cortical neurons in order to produce the motor stimulation. The first one is the average of the firing rate (*FR*) every trial (*k*). We calculate it by integrating all the spikes from each cortical neuron (*n*_*spikes*_) in a window of 1 s (*T*) and dividing it by the number of neurons (*n*_*neurons*_).

(1) $$\begin{array}{l} FR_{k}=\frac{n_{spikes}}{T_{window}}\frac{1}{n_{neurons}} \end{array}$$

By using this feature, we can analyse the frequency of spikes produced by cortical neurons instead of focusing on individual spikes. In parallel, we also use the average and standard deviation of the interspike intervals (ISIs) as the second and third features. We calculate the ISIs of one neuron (n) by checking the time (t) between subsequent action potentials or spikes (i).

(2) $$\begin{array}{l} T_{i}^{n}=t_{i}^{n}-t_{i-1}^{n} \end{array}$$

The average of ISIs of one neuron brings the probable timing of spike, or the interval which a new spike is expected, and the standard deviation of ISIs exploits its temporal coordination or degree of synchrony. For both features, the average value among the cortical neurons are calculated. All those properties are combined as inputs to our DM.

(3) $$\begin{array}{l} \bar{ISIs}=\frac{1}{N}\frac{1}{I}\overset{N}{\underset{n=1}{\sum }}\overset{I}{\underset{i=1}{\sum }}T_{i}^{n} \end{array}$$

(4) $$\begin{array}{l} \sigma_{ISIs}=\sqrt{\frac{1}{N}\frac{1}{I}\overset{N}{\underset{i=1}{\sum }}\overset{I}{\underset{i=1}{\sum }}{\left(T_{i}^{n}-\bar{ISIs}\right)}^{2}} \end{array}$$

Besides that, we used two different properties to evaluate our model under “*parkinsonian”* conditions. First, it is known that *parkinsonian* brains reveal higher synchronicity among neighbouring neurons than healthy brains. And, this behaviour causes a reduction of spike train variability. In order to evaluate our model using this property, we extracted the spike trains of cortical neurons after 10 simulations for each stationary thalamic stimulation and calculated the Coefficient of Variance (CV). The CV, also known as the neuronal variability, can be estimated by using the normalised ISIs distributions. On other words, the standard deviation of ISIs divided by its mean.

(5) $$\begin{array}{l} CV=\frac{\sigma_{ISIs}}{\bar{ISIs}} \end{array}$$

Basically, the ISIs distribution reveals if some intervals of time are repeated more often. The repetition of those intervals helped us to calculate the neuron variability and, as a consequence, to evaluate the model. Another important property used in this work was the pathological beta band activity (13–30 Hz). In order to consider this property, we calculated the Power Spectrum Density (PSD) of different group of neurons (e.g., cortical neurons) using both conditions of the model. The *parkinsonian* condition should produce an enhanced beta oscillation.

#### 6.4.3. Decoding Module

The Decoding Module (DM), described in section 5.2, receives signals corresponding to the dynamics of cortical neurons and translates them into motor stimulation. The robot sensorimotor-loop was designed to produce smooth movements on the robot's arm based on sensory information, therefore we built and trained this module using data extracted from our computational model under *healthy* condition only.

Two different parts of this module were implemented. First, we built and trained a Multi-Layer Perceptron (MLP) network to produce the motor stimulation after three different inputs were extracted from cortical neurons (average firing rate, average ISIs, and standard deviation of ISIs). Then, we implemented a simple smooth filter and a linear function to transform the output data from our neural network in values that are suitable for the robot, respecting the operational range in radians of the robot's joints.

Based on that, a MLP neural network was designed and trained as the main component of the DM. Overall, the motor stimulation generated by this module depends only on the dynamics of cortical neurons. Our MLP was trained using the data extracted from the computational model under *healthy* conditions only.

The main idea was to create a MLP that would inhibit the central oscillators and thus simulate smooth (or *healthy*) robot movements. The resultant movements could be seen as our “ground truth,” i.e., the most accurate measurement available of the robot's smooth (or *healthy*) movements (Marvel et al., 2012; Kondermann, 2013). Hence, without retraining our MLP, we wanted to observe the intervention of the central oscillators in the robot's movements after activating the *parkinsonsian* condition. In this way, when comparing *healthy* and *parkinsonian* conditions, we expected that only the BG-T-C dynamics would change.

Burkhard et al. (2002) state that “oscillatory networks generate a naturally occurring resonance frequency that synchronizes cerebral function, but through an unknown mechanism, they are disinhibited or amplified so as to generate PD or ET tremors.” Therefore, in our initial neurorobotics model, we are assuming that an altered (PD) basal ganglia may interfere with motor programmes (produced, for instance, by the cerebellum) by disinhibiting oscillators that would otherwise not contribute to motor disruption in a *healthy* condition.

In this way, we are not decoding motor commands from our BG-T-C networks, we are instead mimicking the influence of the BG-T-C network on ongoing networks of oscillators that interfere with motor control but that haven't been explicitly modelled. Thus, in our initial model, in the *healthy* condition, regardless of the sensory input, the BG-T-C network should inhibit the influence of such oscillators (modulation values close to 0). Then, in the *parkinsonian* condition, results could be seen as emergent and non intuitive, because the MLP and all other structures, apart from the BG-T-C network, remain exactly the same. This enables us to study the effect of the parkinsonian condition on the inhibition/disinhibition of the central oscillators and its impact on motor behaviour.

In the MLP modelling, we selected the regression mode that could set a predict numeric target instead of creating discrete classes. We used a collection of data obtained after several simulations of our computational model in *healthy* condition to train the entire network. Since we do not want to reproduce any abnormal movement on the robot in *healthy* condition, we trained the network to produce output values equal to zero. This would guarantee that the robot final motor commands translated by the BeM would not be affected by the oscillatory phenomena produced by the central oscillators.

**Figure 8:**
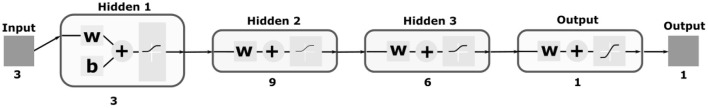
Illustration of the MLP neural network used as part of our DM. The network contains three input neurons which correspond to the properties extracted from cortical neurons; three hidden layers with 3, 9, and 6 neurons, respectively; and one output neuron since we designed the network as a regression model. Log-sigmoid and hyperbolic tangent sigmoid transfer functions were used as presented within the diagrams of the neurons.

The neural network chosen as our DM is composed of three input neurons, two hidden layers with 9 and 6 neurons, respectively, and one output neuron (Figure 8). All neurons contain log-sigmoid transfer functions

(6) $$\begin{array}{l} logsig\left(n\right)=1/\left(1+exp\left(-n\right)\right) \end{array}$$

except by the output neuron which was designed with a hyperbolic tangent sigmoid transfer function

(7) $$\begin{array}{l} tansig\left(n\right)=2/\left(1+exp\left(-2*n\right)\right)-1 \end{array}$$

so it could produce positive and negative outputs. Also, three biases were used to adjust the input data *n* to our model. The network was designed and trained using the Shallow Neural Networks from the Deep Learning toolbox of MATLAB. We used the Gradient descent backpropagation approach to train our network with data collected from our BM after a few trials. The weights generated after our training and the entire architecture can be found in our repository within the DM application.

Two post-processing techniques, smoothing and transformation, were necessary before conveying the motor stimulation to the BeM. Since neural signals are known by their intense oscillation, we decided to apply a mean filter to the output of our MLP network. The designed filter consists of a simple average of five consecutive values (*o*_*t*_, *o*_*t*−1_, ⋯ , *o*_*t*−4_).

(8) $$\begin{array}{l} o_{t}=\frac{1}{5}\overset{4}{\underset{i=0}{\sum }}o_{t-i} \end{array}$$

In this way, only after the fifth output, it is possible to produce the motor stimulation. This was not an issue since the robot spent a few seconds bringing all the packages up and setting itself to its initial pose. Then, a linear mathematical transformation was necessary to adjust those values to a suitable range for the robot's joints in radians.

(9) $$\begin{array}{l} ms_{t}=15*abs\left(o_{t}\right)-0.0015 \end{array}$$

The two parameters of this linear function were manually configured before running the experiments on the real robot.

#### 6.4.4. Behavioural Module

The behavioural module selects the robot's pre-defined *destination poses* after translating sensory inputs, motor stimulation and oscillatory signals to joints trajectories values. Basically, the robot establishes when each movement should be triggered by checking the visual cues from the sensory inputs (positive and negative stimulus) during the task and combining this information to a state machine (SM). Two discrete *destination poses* are established to perform those behaviours. They are used to generate different movements for the robot as presented in Figure 7. Then, the joints angles in radians are adjusted based on the emulated signal (*es*) generated after the modulation of the oscillatory signal (*os*) by the motor stimulation (*ms*).

(10) $$\begin{array}{l} os_{t}=sin\left(\pi t-\frac{\pi }{2}\right) \end{array}$$

(11) $$\begin{array}{l} es_{t}=ms_{t}*os_{t} \end{array}$$

Those operations are executed every second (Δ*t* = 1*s*) during the performance of the task. The oscillatory signal generated from our Central Oscillators was implemented as a sinusoidal wave producing alternated peaks (high and low) at each iteration of the motor loop. Since it is not yet very clear which structures of the brain compose this oscillatory pattern we implemented it as an isolated block of the robot sensorimotor loop (Figure 6).

The state machine (*sm*) selects the robot's action taking into account the oscillatory signal from the Central Oscillators and each joint *j*^*k*^, *k* ∈ {1, 2, 3, 4}, of the arm is updated closing the sensorimotor loop. All the four joints (RShoulderRoll, RElbowRoll, RElbowYaw, RHand) are updated with the same values from our emulated signal.

(12) $$\begin{array}{l} j_{t}^{k}=sm_{t}+es_{t} \end{array}$$

In this work, the motor stimulation is responsible to modulate the oscillatory signals by either suppressing or enhancing the oscillatory signal at each time step. Following findings from the literature on motor modulation of oscillatory signals inherent in the human brain (Burkhard et al., 2002), we designed the system to suppress it under healthy conditions. As a consequence, smooth movements can be executed by the robot throughout the entire task. However, we can not anticipate the robot's behaviour under PD conditions. One can expect that once the *parkinsonian* condition is on, the robot will not be able to modulate the inherent oscillatory patterns anymore.

**Figure 9:**
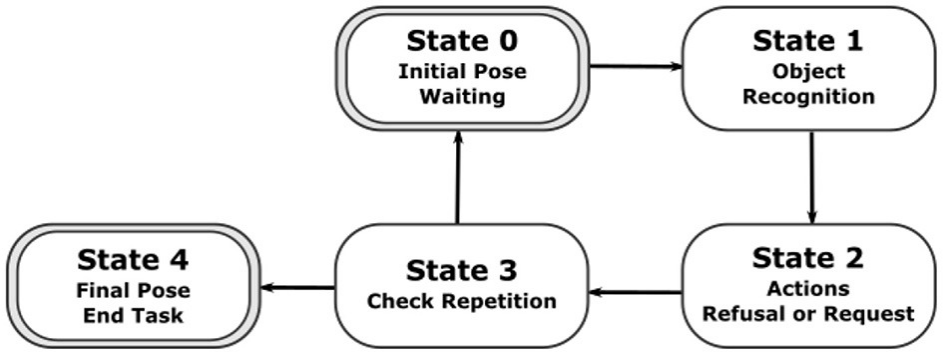
A diagram illustrating the five states of the state machine developed specifically to perform our behavioural task.

In Figure 9, the state machine designed for our behavioural task is illustrated in a simplified way. There are five different states which correspond to different moments of the behavioural task. In the initial state, the robot moves its body to the initial pose and asks someone to allocate one ball in front of him. Once a ball is detected, the state is updated to state 1. In this state, the robot process the sensory inputs in order to recognize the colour of the ball. If the colour is recognised, the state 2 is activated and the action related to the visual cue is executed. When the action completes, the SM moves to State 3 which checks if the number of repetitions has been completed. If it has not, the state 0 is activated again. Otherwise, the SM moves to state 4, which moves its body to the final pose and concludes the task.

#### 6.4.5. Module Integration

ROS provides integration with OpenCV, Netpyne/NEURON, and many other frameworks, libraries, and packages. It is modular and therefore highly versatile. In this work, it simplified the communication between the modules described above. Each module was considered as a different application or node running in parallel during the entire performance of the task. The messages carrying important data, like the sensory neural signal and the motor stimulation, could be transmitted online via topics and services. Besides that, the sensors and actuators of the robot could be easily accessed in high frequency without compromising the task and the dynamics of the BG-C-T system.

## 7. Experimental Results

In this section, we present the experimental evaluations used to assess our neurorobot while performing a simple behavioural task in both conditions of the computational model, *healthy* and *parkinsonian*. Analyses of motor perturbations under the influence of the central oscillators were made to compare its intensity throughout the tasks and while the neurorobot was in both conditions.

### 7.1. Robot Behaviour

**Figure 10:**
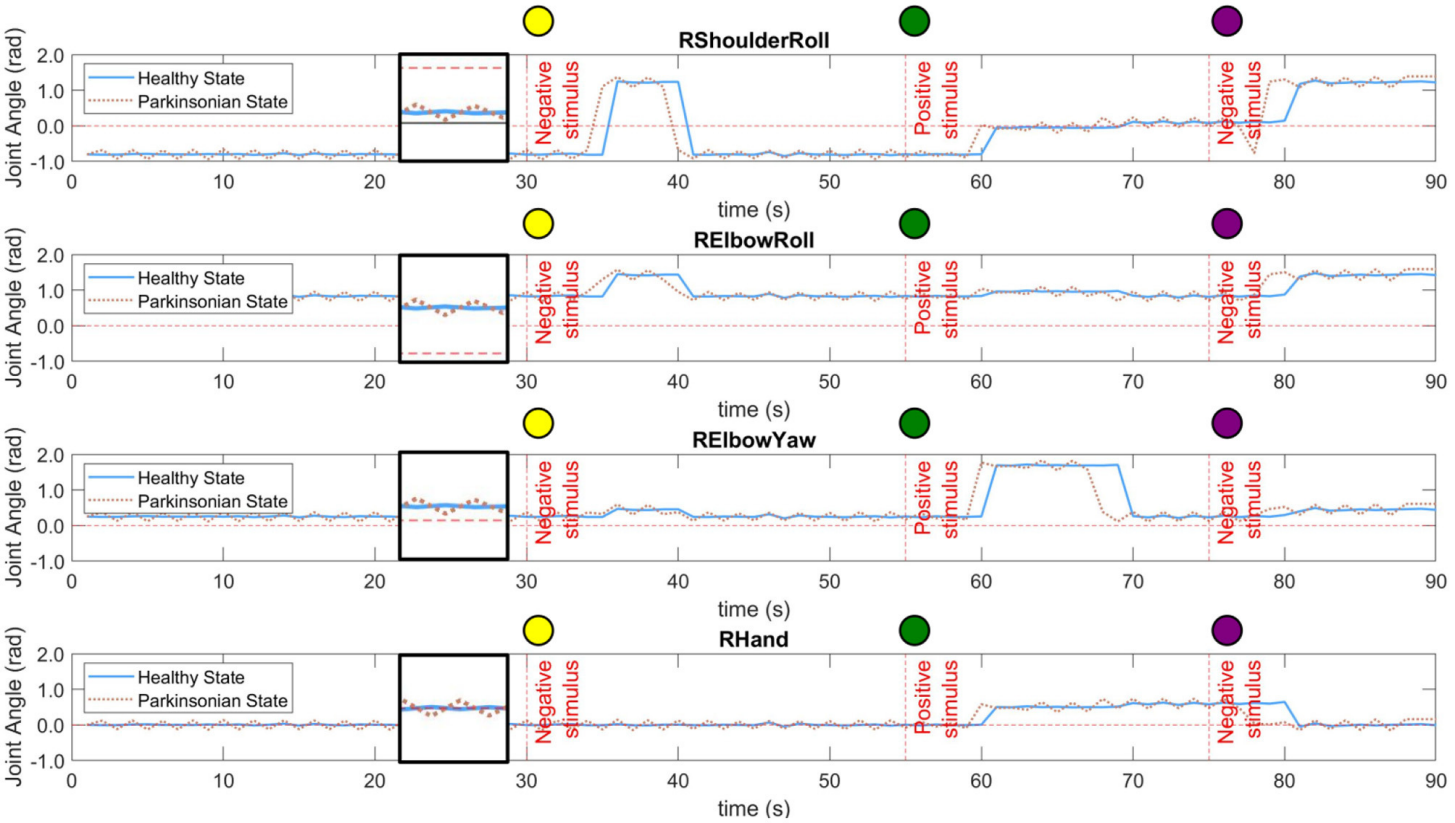
Dynamics of four different joints of the right arm of the robot during the performance of the task. Both conditions of the computational model were tested, *healthy* and *parkinsonian*. The oscillation under the *parkinsonian* condition indicates the production of the tremor symptom in the robot's arm. A small window was created in between 20 and 30 s of each graphic to zoom in on both signals.

Each robot behaviour corresponds to a combination of four different joint angles of the right arm. The joints used in our behavioural task are “RShoulderRoll,” “RElbowRoll,” “RElbowYaw,” and “RHand.” Figure 10 shows the dynamics of each joint during the performance of the entire task for both conditions of the computational model. As described in section 6.2, there are two different experimental scenarios, the rejection and curiosity behaviours. Those two scenarios produce different movements and are represented by different activities in those joints. As mentioned previously, the robot acts a few seconds after the recognition of the object. Because of that, there is a delay on the change of the angles in all cases. Additionally, the interaction with the robot was manually executed which caused reactions of the robot to the same objects not exactly at the same instance of time but yet close to the experimental scenario presented in section 6.3.

In both conditions, one can notice the increase in the intensity of the motor perturbation when the *parkinsonian* condition is activated. The motor perturbation, which could be in a sense analogous to the PD tremor symptom, is reproduced based on subtle oscillations of the joint angles throughout the task. In Figure 10, the dotted line for each joint indicates the tremor pattern that is characteristic in patients diagnosed with PD. However, the frequency and even the amplitude of the movement had to be limited to avoid damaging the joints of the robot. The frequency was adjusted to be the same as the control loop, which is 1 Hz. In this way, every second, the robot could update the joints' angles based on the dynamics of the BeM without compromising the motors. Also, the amplitude of the tremor was constrained based on each joint operational range (SoftBank, 2006).

The trajectory of the arm goes through positive and negative adjustments as already illustrated in Figure 10. During the first 30 s of the experiment, the robot did not interact with any objects which allowed the arm to keep the same position or set of angles. In Table 1, the values present the fluctuation of the arm during this period of time. Since the same values produced by the modulation process are incorporated to all the joints of the arm, we can see that the standard deviation only differs between the condition of the BM. Those two values highlight the disturbance in the robot's arm under *parkinsonian* conditions. By checking those angles in radians, you could think they may not represent a significant change in the trajectory of the robot's arm. But, as you can see in our experiment (https://youtu.be/KEa_2lG8V5s), those extra angles in a joint were enough to create the abnormal movements. Due an increase in the oscillation, the motors needed to move from more distinct positions in the same period of time which caused an increase of velocity and acceleration of the motors.

**Table 1:** The fluctuation of the joints through the first 30 s of the experiment.

**Joints**	**Conditions**	**Operational range (rad)**	**Mean angle (rad)**	**Standard deviation (rad)**
RShoulderRoll	H	−2.0857 to 2.085	−0.7975	0.0145
	PD	−2.0857 to 2.085	−0.7958	0.1260
RElbowRoll	H	0.0349 to 1.5446	0.8325	0.0145
	PD	0.0349 to 1.5446	0.8342	0.1260
RElbowYaw	H	−2.0857 to 2.0857	0.2525	0.0145
	PD	−2.0857 to 2.0857	0.2542	0.1260
RHand	H	Open and Close	0.0025	0.0145
	PD	Open and Close	0.0042	0.1260

*For each joint, it was calculated the mean and standard deviation of its angle in radians. The operational range is also given as a comparative of the robot's maximum and minimum capacity of motion*.

#### 7.1.1. Experimental Scenario 1: Rejection Behaviour

The scenario 1 was performed two times in this experiment. First, when the yellow ball was presented to the robot. And, then, when the purple ball replaced the green one. During the rejection behaviour, the robot moves the right arm close to its torso for a few seconds and, then, returns it to the initial pose. It is visible the execution of this behaviour by the movements of the first two joints in Figure 10 after the first stimulus. They rapidly increase and maintain the angles for a few seconds. The “RElbowYaw” slightly changes its angle and “RHand” keeps in zero which means close hands. When the second negative stimuli (purple ball) is presented, it is possible to see the same behaviour but starting from different states of the joints.

#### 7.1.2. Experimental Scenario 2: Curiosity Behaviour

The second scenario has a completely different dynamics. The robot moves its arm forward, open the hands and wait a few seconds for someone to put the ball on its hand. Then, it grasps and drops the ball into the small container. The self grasping using NAO robot is an extra challenge out of the scope of this work. The curiosity behaviour can be easily observed in Figure 10 after the positive stimulus. The first three joints indicate the movement of the arm forward. The “RHand” opens the hand to hold the object. And, the movement which permit the dropping of the ball can be visualised by the “RElbowYaw” change of angle at 70 s.

### 7.2. The Abnormal Motor Stimulation and the Tremor Symptom

The computational model in *parkinsonian* condition is characterised by the depletion of striatal DA. As explained in section 3, it leads to a functional imbalance of BG circuitry which hampers movement execution. In our experiments, after embedding the model under those conditions in a humanoid robot engaged in a behavioural task, the intensity of the tremor symptom was enhanced.

**Figure 11:**
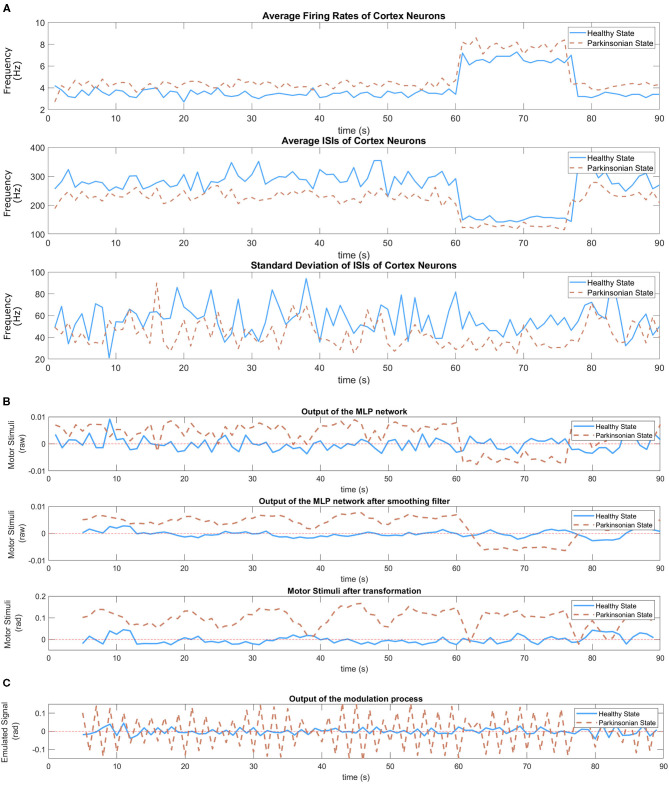
In **(A)**, inputs of the MLP network—average firing rate (Equation 1), average of ISIs (Equation 3), and standard deviation of ISIs (Equation 4) of cortical neurons) during the performance of the task. Two experiments following the same sequence of balls (yellow, green, and purple) were executed, one for each condition of the model. The variation of those stimulus can be easily observed in those graphics. **(B)** Shows the response of the trained MLP network (Figure 8) to the dynamic of cortical neurons under both conditions of the model. A smoothing filter (Equation 8) is applied to the output of the MLP network in order to reduce the oscillation that is common on neural signals. Then, a linear transformation (Equation 9) adjust the value to proper ranges allowing the disturbance to be visible during the execution of the task without compromising the motors of the robot. In **(C)**, the graphic shows the output of the modulation process which corresponds the emulated signal.

In our SML, the DM module produces motor stimulation responsible to modulate the oscillatory signal based only on three different input data (average firing rate, average of ISIs, and standard deviation of ISIs) extracted from cortical neurons. For more details about the DM, please consider reading section 6.4.3. Figure 11A shows those inputs plotted after two experiments, one for each condition of the model. The model under *parkinsonian* conditions produced a higher average firing rate which means more spikes were generated per second (1 Hz). Because of that, there was a reduction in the average of ISIs. In other words, the timing between spikes became shorter. Besides that, the third graphic in Figure 11A shows a slight reduction in synchronicity which means the spikes were generated in a more aleatory manner. During the task, different objects were presented to the robot which produced different levels of excitation in the cortical neurons. For both conditions, the firing rates increased after a positive stimulus which directly produced a certain reduction on the interval between spikes. However, the synchronicity of the neurons seemed to not be affected much.

The distinguishable cortical dynamics allows our trained network to produce different outputs based on the condition of the model. Figure 11B shows the response of the MLP network under both conditions. As it can be seen, abnormal values emerged during the performance of the task under *parkinsonian* condition. We performed a simple filtering process on both output signals since neural activities are known by their intense oscillation. In this way, the second graphic shows clearly the different patterns that was generated. Under healthy conditions, the signal oscillates around the value zero which represents low motor stimulation. Meanwhile, the *parkinsonian* condition brings slightly higher values. Then, we applied a linear transformation as explained in section 6.4.3 to make the motor stimulation suitable for the robot. The generated output represents the motor stimulation in radians produced by the DM during the performance of the task.

As the final step, the motor stimulation modulate the oscillatory signal, generating the emulated signal (Figure 11C), which then tune the angles of the joints defined by our state machine. Basically, for every iteration of the motor loop, a motor stimulus establish the intensity of the motor noise and the central oscillators its signal before adjusting the trajectory of the robot's arm as observed in Figure 10. The tremor symptom is a response from this modulation process between the motor stimulation and the central oscillators.

## 8. Discussion

In this section, we bring some discussions related to the model dynamics, the response of the BG-T-C system to different thalamic stimulation, and other important marks about our SML and PD.

### 8.1. The Model Dynamics

**Figure 12:**
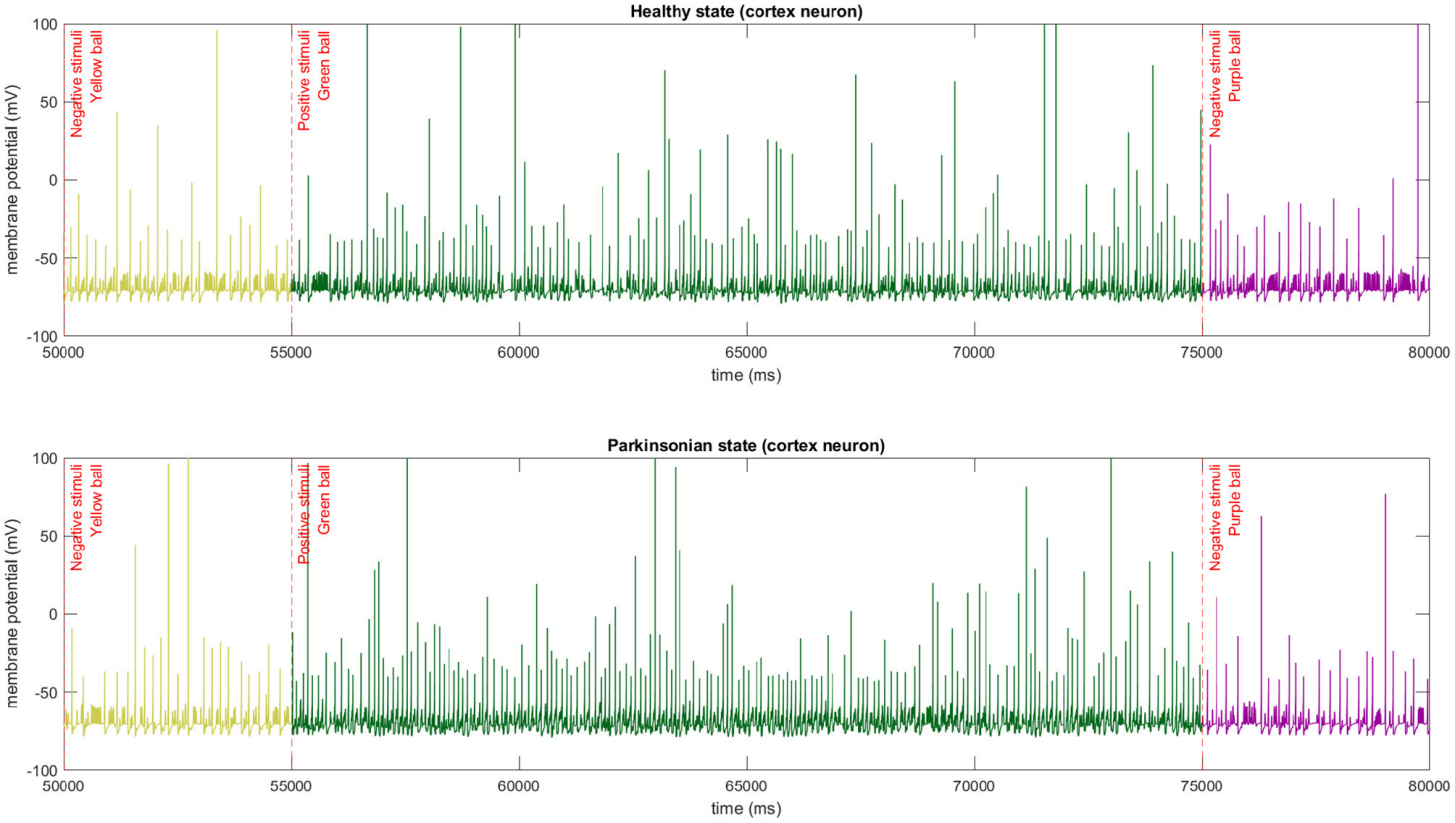
The membrane potential of a single cortical neuron after different stimulus being applied to thalamic neurons. In this experiment, we used the same sequence of stimulus in both conditions of the model. The abrupt change of firing spikes can be easily observed in the graphics.

In our experiments, the colour of the object used to interact with the robot triggers different thalamic stimulus which directly affect the dynamics of cortical neurons (Figure 12). When the green ball is sensed by the robot after a negative or non stimulus the cortical neurons intensify the spiking generation, and the opposite happens when a negative stimuli is presented after a positive one.

**Figure 13:**
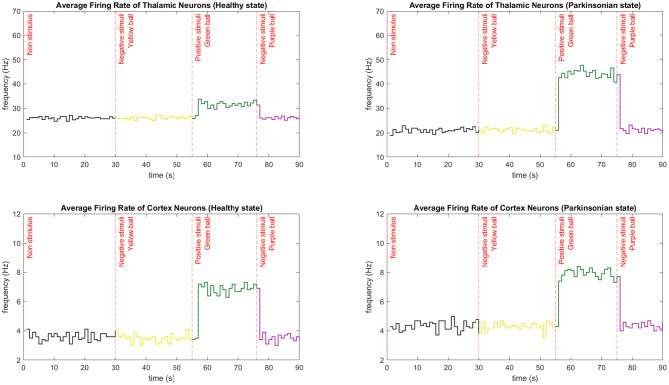
Average firing rate comparison between neurons from CTX and TH during the performance of one entire task. The average firing rate is calculated every second by integrating the spikes from those regions and dividing it by the number of neurons. Three different stimuli were presented to the robot in different moments of the task. The sequence of the colour of the balls in both conditions were yellow (30 s), green (55 s), and purple (75 s). Each graphic shows the exact moment of the transition from a negative to a positive stimuli and vice versa. In those cases, the firing rate rapidly increases and decreases, respectively. The first stimulus maintain the low firing rate since it does not bring any curiosity for the robot. Note that thalamic neurons have a higher firing rate compared to cortical neurons.

By simulating the entire network, we could analyse the dynamics of the robot sensorimotor loop. In Figure 13, the link between the average firing rates of neurons from TH and CTX during the execution of our behavioural task is clear. Every time neurons from TH become more excited, more glutamate is released stimulating even more the motor cortex. And, when TH is inhibited, a reduction of excitation on motor CTX can be also observed. Some other features of the model could be also recognised. For instance, after injecting extra DC current directly to the membrane of the thalamic neurons, there was a significant increase in the number of spikes per second in that region of the brain. In this sense, an external current applied on TH, regardless of the network being on *healthy* or *parkinsonian* condition, contributes to an increase in thalamic firing rate (please refer to section 2.5 from Kumaravelu et al., 2016 for the equations). However, we emphasise that the firing rate in each region results from nonlinear effects. Additionally, due to a cortical network composite of inhibitory (Fast-Spiking Interneurons) and excitatory (Regular-Spiking) neurons, the firing rates of the cortical neurons were considerably lower than the ones from TH. Finally, Kumaravelu et al. (2016) adjusted their model based on data from rats, which shows a higher thalamic firing rate compared to that from CTX. In fact, this is true for primates as well (Van Albada and Robinson, 2009).

Since the initial state of the network does not incorporate external stimulus to TH, a low firing rate is characteristic on both conditions of the computational model. After the first stimuli (experimental scenario 1), the CTX under both conditions kept the average firing rate low since the object (yellow ball) was not excitatory to the robot. It is important to mention that the external stimulus are kept until a new object is placed in front of the robot. Consequently, when the first positive stimulus (experimental scenario 2) was presented, an abrupt increase of the firing rate was detected. For a few seconds, the robot interacted with the green ball, maintaining the high rate. Then, the last object reduced the average firing rate to its initial low level since the object (purple ball) was not excitatory to the robot.

### 8.2. The Thalamic Stimulation

Since the neurorobot depends on different thalamic stimulation to perform the task, we decided to evaluate the injection of a set of different DC currents on thalamic neurons in order to avoid selecting one that could compromise the biological plausibility of the entire computational model. Hence, we believe it is important to elucidate the effect of different thalamic stimulation over cortical neurons before discussing the results about the emerged *parkinsonian* symptom in the robot.

Steriade et al. (1998) once investigated the interplay between those neurons in *in vivo* after repetitive stimuli at around 10 Hz. Based on that, it is known that different thalamic stimuli provoke different responses on cortical neurons. A recent work made a similar experiment but in a PD scope. Tucker et al. (2021) analysed how motor thalamic DBS alters cortical activity by testing different stimulation frequencies, current amplitudes and pulse widths in order to reduce motor symptoms in rats. The authors observed an increase of spike frequencies of cortical neurons after using DBS.

**Figure 14:**
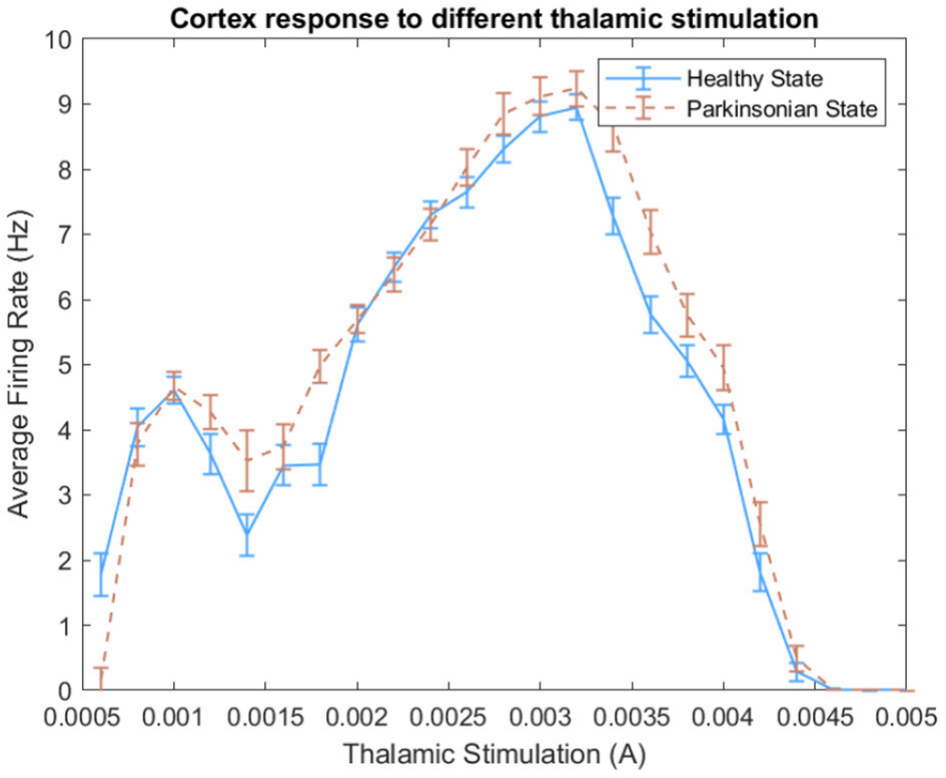
Effect of different thalamic stimulation over cortical neurons. For each thalamic stimulation, we calculated the average firing rate of cortical neurons during an entire experiment and its standard deviation.

In this work, we tested different thalamic stimuli in our BG-T-C circuitry. Figure 14 shows the cortical response to the increase of thalamic stimulation. Since the computational model was designed with excitatory projections from TH to CTX, we expected a proportional increase of the average firing rate in both conditions of the model. However, the model demonstrated a non-linear proportionality within two ranges of thalamic stimulation. For instance, a noticeable peak indicates that the model changes the cortical dynamics after reaching 3.2 mA. Then, it rapidly reduces the average firing rate, reaching zero at 4.6 mA approximately. Besides that, a slight increase of spikes can be observed when the *parkinsonian* condition is activated for several ranges of thalamic stimuli.

Based on that, we choose different pairs of DC currents and ran some analyses. We selected values that could respect PD features and generate distinguishable dynamics on cortical neurons. We assessed the model following some PD features like spike train variability of neighbouring neurons and anomalous beta oscillation. Then, we decided to inject 1.2 mA of DC current on thalamic neurons every time the robot sense a negative stimulus and 3.5 mA for the positive stimulus.

**Figure 15:**
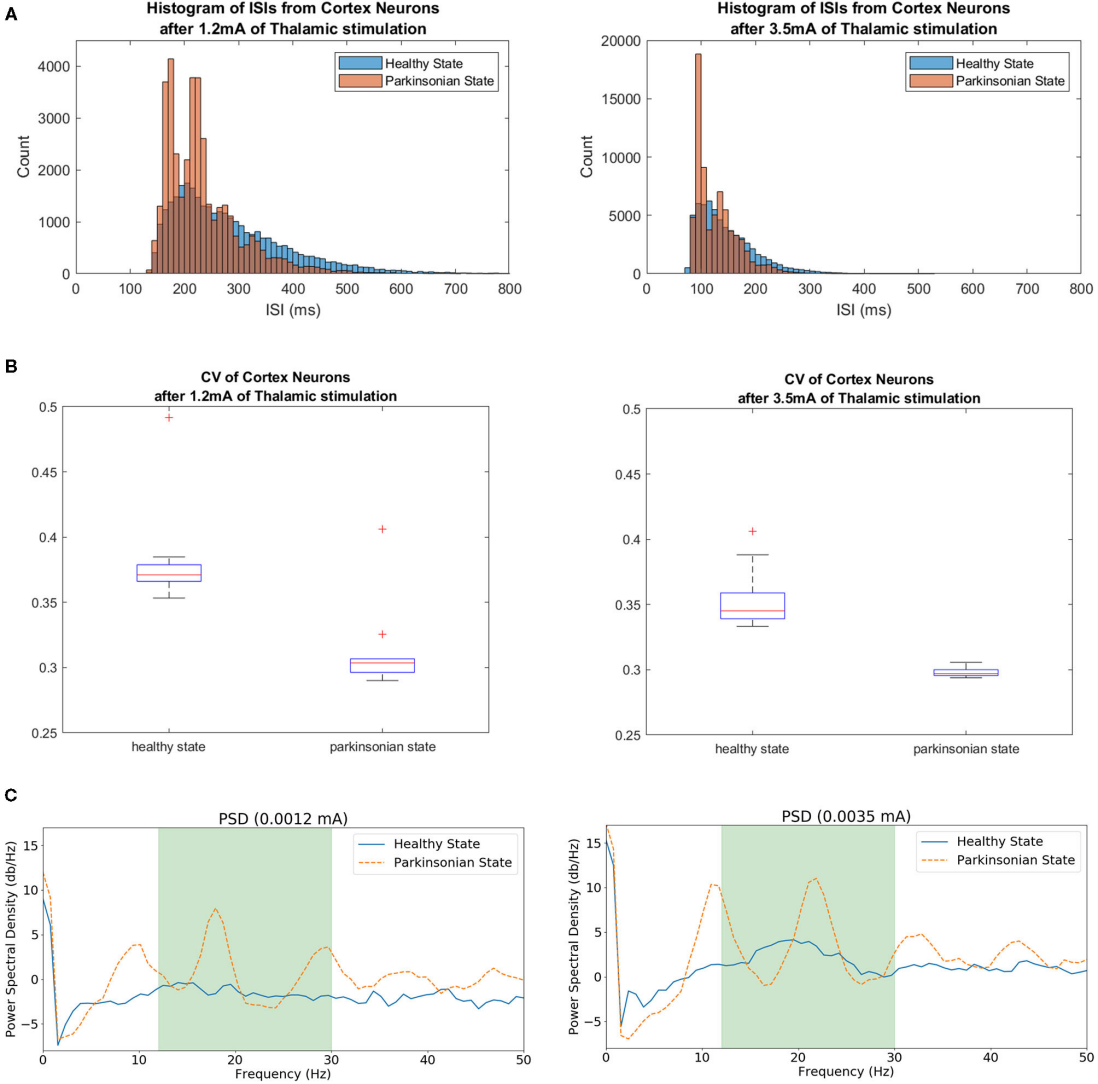
In **(A)**, histograms of ISIs from simulations under different thalamic stimulation (1.2 and 3.5 mA) in both conditions of the model. The range for each histogram was adapted since a higher thalamic stimulation increases the neuronal activity from CTX, producing even more spikes. A higher concentration of the same intervals between spikes indicate the reduction of variability when the *parkinsonian* condition is activated in both scenarios. **(B)** Shows the coefficient of Variance of cortical neurons after two thalamic stimulation. The variability was tested in both scenarios for both conditions of the model. The box plots show a reduction of variability when the *parkinsonian* condition is activated. It also shows a bigger concentration of ISI values. **(C)** Exhibits Power Spectrum Density (PSD) of cortical neurons under 1.2 and 3.5 mA of thalamic stimulation. Both conditions of the model were tested. The green area indicates the beta band range (13–30 Hz). The peaks within the range shows the enhance in beta activity detected after activating the *parkinsonian* condition.

In order to evaluate the variability, we built histograms of ISIs of cortical neurons under different thalamic stimulation using both conditions of the model. Figure 15A shows the resulting non-symmetric distributions. The ISI values produced right-skewed distributions that are characterised as having a higher concentration on its left side of the curve and a longer tail on the other side. The intervals with a higher concentration can be visualised around 200 ms for the 1.2 mA scenario and 125 ms for the 3.5 mA scenario. In both cases, under the *parkinsonian* condition, the peaks indicate a clear reduction in variability. On other words, the neurons produce more often spikes with same intervals.

Another characteristic that can be observed from both distributions is the increase of the number of ISIs. As exhibited in Figure 14, 3.5 mA of thalamic current generates more spikes on cortical neurons. As a consequence, it reveals more intervals between spikes which obviously increases the number of ISIs. Because of this feature, it can be observed that one peak reach values close to 4,000 ISIs while the other goes over 18,000. Despite this noticeable variation, the graphics maintains the shape of the distributions in both scenarios and conditions.

In order to quantify the variability, the normalised version of the standard deviation of ISIs known as CV was used. Figure 15B makes evident the reduction of variability of cortical neurons after activating the *parkinsonian* condition in both scenarios. This feature relates to an increase of synchronicity between neighbouring neurons.

The other PD feature investigated in this work was the enhance of beta activity in the corticothalamic-basal ganglia network. As it can be seen in Figure 15C, the beta band was enhanced after activating the *parkinsonian* condition of the model in both scenarios (under 1.2 and 3.5 mA of thalamic stimulation). The peaks in both graphics show a significant increase of activity.

### 8.3. Final Considerations

The embodiment of autonomous neural systems that are based on the structure of animal brains is yet a young field. However, it has already brought important discussions on neural models and their applications. It permits the conduction of experiments in a repeatable manner without bringing any ethical constraint. As discussed in section 4, only a few works have embedded computational models of neural disorders in robots. We could observe that the majority of the researches in neurorobotics focus on common robotics problems, like navigation and decision-making, instead of building tools to support neuroscientists with their findings on neural disorders.

In this work, we embedded a composite model of the BG-T-C system in a real humanoid robot to perform movements based on sensory information. The realistic biophysical model allowed the robot to replicate the interplay between neurons from different regions of the brain during the performance of the task without demanding a high computational power. With our neurorobot, we could stimulate thalamic neurons and analyse its response over the entire model using different approaches, like mean firing rate, spike train variability, and even beta oscillation activity. Several experiments were executed without compromising our robot and the model. We choose thalamic neurons to be injected with external DC currents since they control the flow of information that are transmitted to cortical neurons. However, some other works have decided to explore other regions of the brain (Kim et al., 2017; Mulcahy et al., 2020). In our experiments, the robot was able to sense the environment, process the information and act respecting the same dynamics observed on data sets from animal's brains.

When activated the “*parkinsonian”* condition, one of the PD symptoms was reproduced during the performance of the task. The combination of different frameworks and libraries made possible to embed the PD model in a robot engaged in a behavioural task. In this way, the sensory information collected from the environment had an important role in our SML which allowed the robot to take its action with incorporated motor noise. Since this work represents the first step toward a realistic neurorobotics model of PD, the main idea was to develop those components in a modulated way, allowing future replacements. Five different modules were created. Simple updates can be made on each module and, even, a complete different implementation. However, the communication between modules must be respected in order to maintain the data flow through our SML. ROS made possible not only the communication between those applications but also the interaction with the robot's hardware.

It is important to highlight that for both conditions, *healthy* and *parkinsonian*, the entire SML used was the same except by the conditions applied to the BM, and the motor stimuli generated was constantly used to modulate the oscillatory signal to incorporate different intensities of motor tremor in the robot motion. However, the *parkinsonian* condition produces values that cause more perturbation to the robot's upper limbs movement, while the *healthy* condition produces minimum-variance trajectory or unnoticeable tremor similar to what happens in healthy animals (Harris and Wolpert, 1998). Hence, the major change between the conditions of our neurorobotics model is the amplitude of the tremor.

This research produced a novel tool that can be used in several contexts, not only PD. The computational model of PD represent the biophysical state of an entire SML. Each module designed within this loop can be modified and adapted to different scenarios and even completely different applications. Neuroscientists could generate new hypotheses relate to other neural disorder that is involved to the BG-T-C system. And, even use the model to understand the dynamics of those neurons respecting the different motor pathways.

Prescott et al. (2006) once questioned which would be the most suitable interpretation of the inputs and output of computational models of the brain. Authors explain that is common to associate “sensory” signals as input and “motor” signals as output of those models but it might be not the most appropriate representation. We are searching for a more biologically plausible mechanism to coordinate movements in a robot.

In neuroscience, some works investigate the mechanical properties of muscles (Harris and Wolpert, 1998). They bring interesting research questions like how trajectories of arm movements can be performed smoothly and how much control the central nervous system has in this scenario. By using the same theory of control systems, we assume there is an autonomous mechanism responsible for optimising the integration of those movements. For instance, a mechanism capable of generating the correct amount of stimulus for each muscle in small instances of time during the entire trajectory. In this context, we will adapt our SML to new behavioural tasks that will allow us to focus more on the motion of the arm. The BM will be evolved with new components, a novel DM will be designed and implemented allowing the reproduction of the motor pathways between brain and muscles, and a new BeM will allow the execution of more complex tasks.

Another point that is important to discuss is the level of realism of the humanoid robots used in our experiments. Since a more realistic DM will be built, it will be important to move the experiments to a more complex scenario where the robot can reproduce movements that resemble more humans. In this way, we will use the iCub robot instead of keeping using the NAO robot for the upcoming experiments. Movements that are commonly used in therapy sessions with PD patients will be replicated by the iCub robot. And this task will allow us to asses our model by comparing its movements to data collected from real patients under same activities.

Finally, the origin of the PD tremor symptom is yet elusive. Many hypotheses have been created by different research groups but no consensus is in sight. By finding how this neural disorder is triggered and developed, it improves the chances of 1 day finding the cure. Here, we investigated the BG-C-T system and observed the cortical neurons dynamics through different thalamic stimulation and different conditions of the model. The emerged symptom was a result of a combination of different properties extracted from cortical neurons and the oscillatory signal. It might be a naive approach initially. But this is only our first step toward the PD neurorobotics model. We understand the complexity of this problem and we hope that our research outcome might become a new tool for neuroscientists in their further works.

## 9. Concluding Remarks and Future Work

In this work, we present an initial neurorobotics model of Parkinson's Disease (Neuro4PD). The proposed model showed that it is possible to reproduce motor movements with different levels of perturbation based mainly on the dynamics of the cortex and the modulation of oscillatory phenomena inherent in the human brain, both in *healthy* and *parkinsonian* conditions.

It was possible to observe that the perturbation on the movement can be in a sense analogous to the tremor symptom, that is characteristic on PD patients. Our behavioural task made it possible for us to focus more on the aspects of PD instead of motor control. However, the robot sensorimotor loop is yet a simple representation of how the nervous system interacts with the upper limbs.

The Neuro4PD is a neurorobotics model that represents a new platform with a strong potential for numerous applications. For instance, it can be used to shed light into PD research and also to support investigation on the BG-C-T circuitry within a sensorimotor loop. Other models could also be incorporated to the circuitry allowing others functionalities to be studied. Moreover, a representation of the cerebellum and amygdala could be linked to the model in order to incorporate the concept of memory. As a consequence, even more complex behavioural tasks could be explored in this context and also other neural disorders.

Future work involves moving to a computational model of the brain that is more closer to the human brain in terms of anatomy and features of PD. In this work, we used a model that was built using data from a rat brain after an unilateral infusion of the neurotoxin 6-hydroxydopamine (6-OHDA). There are some similarities within the sensorimotor loop of rats and humans but better representations can be found on other species. For instance, primates share more genetic similarities to humans. Not only the anatomy of the brain but also the neuropathophysiology of PD. We believe that a more realistic computational model of the brain based on primates will improve the realism of our work and increase the contributions to our community (Ranieri et al., 2020).

Another natural follow-up to our work is to enhance motor control by directly decoding motor commands from cortical activity, possibly including different striatal inputs related to action-selection mechanisms. This improved approach would coexist with our current model, given that they relate to different aspects of motor control.

Overall, by further understanding the interplay between neural dynamics, physical embodiment and environmental factors, we believe that even more complex brain-based robots could be developed unveiling new robot applications based on recent neuroscience findings.

## Data Availability Statement

The original contributions presented in the study are included in the article/Supplementary Material, further inquiries can be directed to the corresponding author/s.

## Author Contributions

JP is the main author and contributed to all aspects of this study. PV and RM have contributed to the approach and methodology design, overall text and content. MA have contributed to the overall text and content. CR, RR, and FB provided crucial feedback on manuscript. All authors contributed to the article and approved the submitted version.

## Conflict of Interest

The authors declare that the research was conducted in the absence of any commercial or financial relationships that could be construed as a potential conflict of interest.

## Footnotes

^1^This work is part of a multidisciplinary project, the Neuro4PD: Neurorobotics Model of Parkinson's Disease. Its main goal is to gain further insights into the mechanisms of PD by combining neuroscience, machine learning and robotics expertise from Brazil and the UK. The project is funded by the Newton Fund and Royal Society in the UK (https://www.macs.hw.ac.uk/neuro4pd/).

^2^Somatotopic organisation means that each part of our body corresponds to a specific location within a structure in the central nervous system.

^3^Or, in the rat, the homologous entopeduncular nucleus, EP.

^4^Each module is available on a github repository (https://github.com/jhielson/Initial_Neurorobotics_Model_of_PD) with commented material.

^5^In this work, as a proof of concept, we focused on moving only the right arm. The left arm was kept still so it does not compromise the experimental results.
