# Supplementary material for: Neuro4PD: An Initial Neurorobotics Model of Parkinson's Disease
Source: Front Neurorobot. 2021 Jul 1;15:640449. doi: 10.3389/fnbot.2021.640449 (PMC8283825; doi:10.3389/fnbot.2021.640449)
Supplement: Supplementary file 3 [file Data_Sheet_2.PDF]

Supplementary Material

The BG-T-C system that was incorporated into our Brain Module of the robot sensorimotor loop replicates the same neural dynamics observed on Kumaravelu et al. (2016). It was implemented by Romano et al. (2020) which adapted it to an open-source Python package. The following table exhibits the equations of the membrane potential of the neurons from each region of the brain. For more details on those equations please consult Kumaravelu et al. (2016).

Model Neurons	Membrane Potential
Cortex (CTX):	
- regular spiking (RS)	$\frac{\mathrm{dv_{rs}}}{\mathrm{dt}} = 0.04 * v_{rs}^2 + 5 * v_{rs} + 140 - u_{rs} - I_{ie} - I_{thco}$
- fast-spiking inhibitory interneurons (FSI)	$\frac{\mathrm{dv_{fsi}}}{\mathrm{dt}} = 0.04 * v_{fsi}^2 + 5 * v_{fsi} + 140 - u_{fsi} - I_{ei}$
Striatum (Str)	$C_m \frac{\mathrm{dv_{Str}}}{\mathrm{dt}} = -I_l - I_K - I_{Na} - I_m - I_{gaba} - I_{costr}$
SubThalamic Nucleus (STN)	$C_m \frac{\mathrm{dv_{STN}}}{\mathrm{dt}} = -I_{Na} - I_K - I_a - I_L - I_T - I_{Cak} - I_l - I_{gesn} - I_{cosn,ampa} - I_{cosn,nmda} + I_{dbs}$
Globus Pallidus externa (GPe)	$C_m \frac{\mathrm{dv_{GPe}}}{\mathrm{dt}} = -I_l - I_K - I_{Na} - I_T - I_{Ca} - I_{ahp} - I_{snge,ampa} - I_{snge,nmda} - I_{gege} - I_{strgpe} + I_{appgpe}$
Globus Pallidus interna (GPi)	$C_m \frac{\mathrm{dv_{GPi}}}{\mathrm{dt}} = -I_l - I_K - I_{Na} - I_T - I_{Ca} - I_{ahp} - I_{sngi,ampa} - I_{gegi} - I_{strgpi} + I_{appgpi}$
Thalamus (TH)	$C_m \frac{\mathrm{dv_{Th}}}{\mathrm{dt}} = -I_l - I_K - I_{Na} - I_T - I_{gith} + I_{appth}$

Table S1. Membrane potential of different neurons per region of the brain. Those equations describe the neural dynamics observed on Kumaravelu et al. (2016). For further information please consult their work.

REFERENCES

Kumaravelu, K., Brocker, D., and Grill, W. (2016). A biophysical model of the cortex-basal ganglia-thalamus network in the 6-ohda lesioned rat model of parkinson's disease. *Journal of computational neuroscience* 40. doi:10.1007/s10827-016-0593-9

Romano, M. R., Moioli, R. C., and Elias, L. A. (2020). Evaluation of Frequency-Dependent Effects of Deep Brain Stimulation in a Cortex-Basal Ganglia-Thalamus Network Model of Parkinson's Disease. *Annu Int Conf IEEE Eng Med Biol Soc* 2020, 3638–3641
